# Current needs for the improved management of depressive disorder in community healthcare centres, Shenzhen, China: a view from primary care medical leaders

**DOI:** 10.1186/s13033-019-0300-0

**Published:** 2019-06-28

**Authors:** Kendall Searle, Grant Blashki, Ritsuko Kakuma, Hui Yang, Yuanlin Zhao, Harry Minas

**Affiliations:** 1 Global and Cultural Mental Health Unit, Centre for Mental Health, Melbourne School of Population and Global Health, University of Melbourne, Parkville, VIC 3010 Australia; 2 Nossal Institute for Global Health, The University of Melbourne, Melbourne, VIC 3010 Australia; 3 London School of Hygiene and Tropical Medicine, London, WC1E 7HTE England UK; 4 Monash Institute for Health & Clinical Education, School of Primary Health Care, Monash University, Notting Hill, VIC 3168 Australia; 5 Faculty of Education, Queen’s University, Kingston, ON Canada

**Keywords:** Mental health, Mental, neurological and substance use disorders (MNS), Depressive disorder, Depression, Common mental disorders, Mental illness, Mental Health GAP Intervention Guide (mhGAP-IG.v2), Primary care, Community healthcare centres, Health system reform, Shenzhen, China, Theoretical Domain’s Framework (TDF), Barriers, Enablers, Diagnosis, Assessment, Management, Follow-up, Clinical practice

## Abstract

**Background:**

The prevalence of depressive disorder in Shenzhen is higher than for any other city in China. Despite national health system reform to integrate mental health into primary care, the majority of depression cases continue to go unrecognized and untreated. Qualitative research was conducted with primary care medical leaders to describe the current clinical practice of depressive disorder in community healthcare centres (CHC) in Shenzhen and to explore the participants’ perceptions of psychological, organizational and societal barriers and enablers to current practice with a view to identifying current needs for the improved care of depressive disorder in the community.

**Methods:**

Seventeen semi-structured, audio-recorded interviews (approx. 1 h long) were conducted in Melbourne (n = 7) and Shenzhen (n = 10) with a convenience sample of primary care medical leaders who currently work in community healthcare centres (CHC) in Shenzhen and completed any one of the 3-month long, Melbourne-based, “Monash-Shenzhen Primary Healthcare Leaders Programs” conducted between 2015 and 2017. The interview guide was developed using the Theoretical Domain’s Framework (TDF) and a directed content analysis (using Nvivo 11 software) was performed using English translations.

**Results:**

Despite primary care medical leaders being aware of a mental health treatment gap and the benefits of early depression care for community wellbeing, depressive disorder was not perceived as a treatment priority in CHCs. Instead, hospital specialists were identified as holding primary responsibility for formal diagnosis and treatment initiation with primary care doctors providing early assessment and basic health education. Current needs for improved depression care included: (i) Improved professional development for primary care doctors with better access to diagnostic guidelines and tools, case-sharing and improved connection with mentors to overcome current low levels of treatment confidence. (ii) An improved consulting environment (e.g. allocated mental health resource; longer and private consultations; developed medical referral system; better access to antidepressants) which embraces mental health initiatives (e.g. development of mental health departments in local hospitals; future use of e-mental health; reimbursement for patients; doctors’ incentives). (iii) Improved health literacy to overcome substantive mental health stigma in society and specific stigma directed towards the only public psychiatric hospital.

**Conclusions:**

Whilst a multi-faceted approach is needed to improve depression care in community health centres in Shenzhen, this study highlights how appropriate mental health training is central to developing a robust work-force which can act as key agents in national healthcare reform. The cultural adaption of the depression component of the World Health Organisation’s mental health gap intervention guide (mhGAP-IG.v2) could provide primary care doctors with a future training tool to develop their assessment skills and treatment confidence.

**Electronic supplementary material:**

The online version of this article (10.1186/s13033-019-0300-0) contains supplementary material, which is available to authorized users.

## Background

China accounts for 17% of the global burden of mental, neurological and substance use disorder (MNS) [1]. Within China, the disease burden attributed to MNS is over 10 million disability adjusted life years (DALYs) and is expected to increase by 10% by 2025 due to China’s rapidly aging population [1]. Each year, over 50 million people in China (3.6% of the population) suffer from depressive disorder [2], one of the most prevalent MNS disorders. Depressive disorder is characterized by the “presence of sad, empty, or irritable mood, accompanied by somatic and cognitive changes that significantly affect the individuals’ capacity to function” [3]. Depressive disorder is strongly associated with suicide, China’s 8th most frequent cause of death and accounts for 26% of suicides globally [4]. Depressive disorder also exerts an indirect economic burden on China in excess of US$6.3 billion per annum [5]. Despite its far-reaching effects at the community, national and global level, and the availability of cost-effective mental healthcare [6, 7], the vast majority of depression cases in China remain undiagnosed and untreated [8].

Shenzhen, located in Guangdong Province, Southern China, was established as the first of China’s Special Economic Zones in 1980. Its rapid urbanisation, from a fishing village to a high-tech manufacturing and global centre of commerce [9], has been accompanied by large-scale internal migration [10] such that its vast, youthful, migrant work-force has doubled Shenzhen’s official residential population [11] to over 20 million. This radical change in population demographics has had both a positive and negative impact on the health of Shenzhen’s communities [10]. The prevalence of mental disorders is higher in Shenzhen than in any other city in China [12, 13] and the prevalence of depression in key working populations could be as much as 5–8 times higher than the national estimates [14, 15].

The barriers to depression care in China are becoming increasingly well described with regards to patient, societal and health system influences. Persons suffering from depression tend to avoid seeking treatment due to: poor general health literacy and depression-specific awareness; traditional cultural values of “face” and the fear of stigma [16–18]; perceived high cost of healthcare, inequitable insurance coverage and concerns about the impact of poor health on employment opportunities [19–21]; and low level of trust in and respect for doctors’ treatment abilities [22–24]. At the health system level, doctors are in short supply. There are only 1.4 general practitioners per 10,000 people in China [25] (compared to 9.7 general practitioners per 10,000 people in Australia [26]) and 1.7 psychiatrists per 100,000 people in China [27] (compared to 11.87 psychiatrists per 100,000 population in high income countries) [28]. Furthermore, depressive disorders (unlike other psychosis), have only recently achieved national policy consciousness, [18, 29]. Most primary care doctors have received limited mental health training [30] and lack the diagnostic skills needed to detect cases that are frequently masked by somatic symptom presentation [17, 31, 32], particularly within consultation times that may be as short as four minutes [22]. Doctors find themselves increasingly demoralized by their poor working conditions [22] and aspects of diagnosis and patient quality of care may be compromised.

Western healthcare systems predominantly manage and treat depression in primary care. This approach derives from evidence that most patients visit their doctor during the course of a depressive episode [33], and that doctors, (due to their regular patient contact, knowledge of family background [34] and availability of general practice validated depression screeners [35]), can optimally detect depression and manage both psychosocial and drug treatment [6]. Likewise, since 2011, the Chinese government has committed to extensive National Health System reform to reduce the burden on hospital-based medicine and to expand the role of primary care clinics as the first point of contact for non-acute and chronic conditions [36, 37]. According to these initial reforms, primary care physicians were given responsibility for basic clinical care and public health service whilst hospital specialists provided more formal diagnosis and initiation of drug treatments.

However, the role of the primary care physician is rapidly being redefined. China’s Second Mental Health Work Plan 2015–2020 formally recognized, for the first time, the increasing burden of common mental disorders and tasked primary care clinics with “significantly enhancing” their prevention and treatment activities in the community [38]. Furthermore, recent plans for “Healthy China 2030” [39], and global recommendations for ideal mental health service components in low-resource settings [40] suggest that primary care physicians in China will need to become key initiators of evidence-based drug therapies for mental health conditions in the future. The Shenzhen Medical Training Centre, has rapidly absorbed and acted upon these national calls for change in healthcare practice. In particular, through its pursuit of training-up local doctors to high standards through international capacity building initiatives such as the Monash-Shenzhen Primary Healthcare Leaders Program^1^ [41, 42].

The objectives of this study were to explore the current needs for the improved management of depressive disorder in community healthcare centres (CHC) in Shenzhen by: (i) describing the current clinical practice of depressive disorder in CHCs (ii) identifying psychological, organizational and societal barriers and enablers to current practice based on the knowledge, experience and perspectives of primacy care medical leaders.

Primary healthcare research is a relatively new field in mainland China. So too is the shift in focus towards considering mental health as a responsibility of primary care practitioners. Currently, to our knowledge, there are no other China-based studies that investigate depressive disorder treatment practice needs from the perspective of community-based doctors and also use an established qualitative research framework which considers key constructs of behavioral change.

## Methods

### Sample

Seventeen semi-structured interviews were conducted in Melbourne (n = 7) and Shenzhen (n = 10) with a convenience sample of primary care medical leaders who currently work in community healthcare centres in Shenzhen and who have completed any one of the 3-month long, Melbourne-based Monash-Shenzhen Primary Healthcare Leaders Program conducted between 2015 and 2017.

### The Monash-Shenzhen Primary Healthcare Leaders Program

Enabled the researchers to both identify eligible participants and provided a vital contact site for participant engagement. The program was established in 2011, as an educational collaboration between Monash University and The Shenzhen Medical Training Centre, to provide capacity building for future leaders of Shenzhen’s primary healthcare sector. Due to the stringent entry requirements (successful applicants must demonstrate academic excellence, English proficiency at examination and leadership qualities assessed by a competitive interview process) and associated government funded award, graduates from of this course are highly regarded by the Shenzhen primary care community as future healthcare leaders. Their views are invaluable to this research, as they represent early adopters of healthcare reform and reflect the forefront of primary healthcare opinion.

### Recruitment and fieldwork

Three cohorts of trainees (28 doctors) were invited (via Wechat) to a face-to-face group information session provided in Melbourne, at the end of each training course. 61% agreed to participate. The main reason communicated for non-participation was “difficulty to schedule an appointment” during the available fieldwork time. One-hour long, audio-recorded interviews with participants took place in Melbourne (December 2017) and in Shenzhen (January 2018). They were conducted by one of two native Mandarin language interviewers (with prior experience of social research) accompanied by the researcher. Each interviewer was also responsible for transcribing verbatim and translating into English their individual quota of interviews. Both interviewers were fully briefed of the study background and objectives and were closely involved with all stages of research requiring translation.

### Interview guide

The interview guide (approx. 1 h long) was developed using the Theoretical Domains Framework (TDF) [43] (see Additional file 1) which brings together psychological and organizational theories on behavior change into one framework consisting of 14 domains. It has been used by a variety of healthcare professionals and across disease settings including amongst community-based healthcare doctors in Shenzhen [44–46] to inform the development of interventions that require both users and a wider organization to change their current approach to a prevailing concern.

In this research, the application of the TDF ensured that multiple lenses of enquiry were used to explore not just what doctors do when they see a patient with depressive disorder, but why they do it. It gave equal weight to investigating how a doctors’ personal belief system/professional identify and external factors (e.g. health system; society at large) may shape their response to providing mental health care. This format facilitates the emergence of perceived challenges and opportunities at multiple levels to help inform areas for future improvement to depression care. Specifically, the framework was applied as follows:

i. *Current practice* (Domains 1–5: knowledge; optimism; beliefs about consequences; memory, attention and decision processes; and skills) were directed at determining the current “status quo” of depressive disorder within doctors’ clinics, with regards to doctors’ awareness of the condition and symptom profile, their standard consultation response, and their understanding of patient outcomes and the receipt of care. Optimism relates to the mental health treatment gap (i.e. how optimistic doctors are that identified patients will receive care). In order to bench-mark against the mhGAP-IG, particular care was taken to explore assessment, management and follow-up aspects of care. In addition, the guide collected information on what governance and guidelines are in place and the type and level of skills currently being used in primary care to manage patients.
ii. *Doctors’ psychological response to providing care* (Domain 6–8: beliefs about capabilities; social/professional role and identity; emotion) asked the doctors to look introspectively; to reflect upon their professional role and their personal abilities, limitations or emotional needs which may influence the receipt of depression care.
iii. *External influences* (Domain 9–10: environmental context and resources; social influences) were concerned with external influences. In this case, the specific work environment of the doctor’s clinic and the broader social backdrop with governs the thinking of society at large as well as that of the surrounding local community.
iv. *Enablers and future needs* (Domains 11–12: behavioral regulation; reinforcement) opens-up doctors thinking to what they may already have (i.e. enablers) or need to improve care for patients with depressive disorder.
v. *Desire to change* (Domains 13–14 intentions; goals) can be likened to a doctor’s barometer of how important this issue is for them currently and what actionable goals are in place (if any) to succeed in this area.

### Patient profile

Interviews were initiated by presenting doctors with a patient profile (also in Mandarin Chinese) [47] with symptoms consistent with a Diagnostic and Statistical Manual 5 (DSM-5) diagnosis of depressive disorder and requiring antidepressant medication by mhGAP-IG standard protocol for treatment. This approach sought to avoid any potential cross-cultural or translational misunderstandings regarding the terminology for depressive disorder and ensured a focused discussion (i.e. on depressive disorder not more severe disorders such as psychosis).

### Analysis

Directed content analysis^2^ [48, 49] was performed using Nvivo11 software. Core belief statements were first grouped using the fourteen TDF domains, then sub-themes were identified and tagged for consideration as sub-codes (KS). Theoretical saturation (i.e. no more emergent themes were identified with additional data) was achieved within this sample size as is consistent with literature directed at establishing the ideal sample size for qualitative research [50]. Core belief statements and supporting themes were back translated to ensure cultural nuances were maintained (YZ) and the code frame discussed with the interviewer team prior to data-coding. 10% of coding was back-checked by a bilingual interviewer (YZ). Final supporting quotes were also back-translated for quality.

## Results

### Demographics

**Fig. 1:**
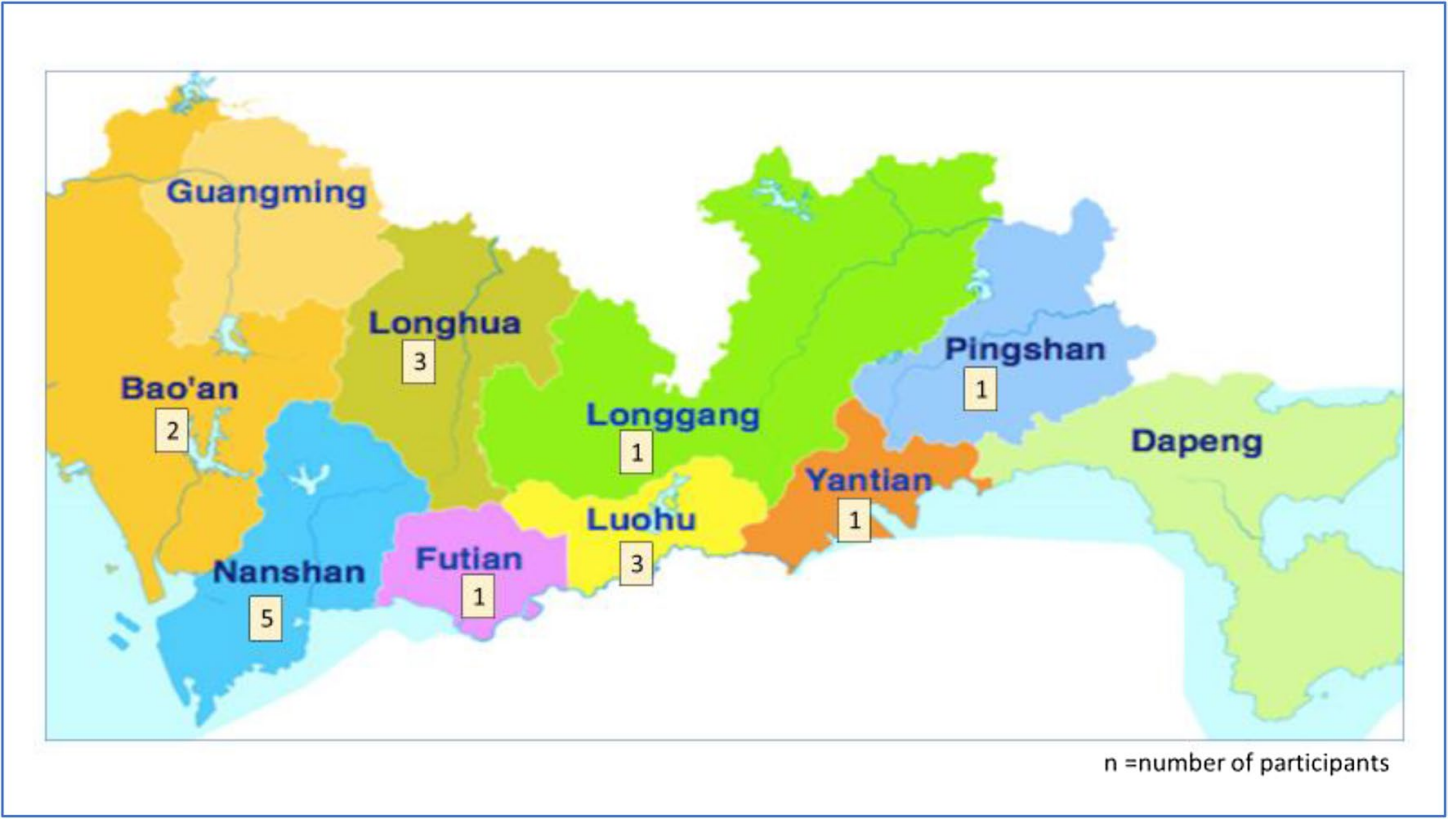
Location of CHC where participants are based according to administrative districts in Shenzhen (n = 17)

A total of 17 primary healthcare leaders, 11 females and 6 males, participated in the study. All held senior management positions at clinic or district level (i.e. senior doctor, assistant clinic director; clinic director; district clinic director) and had an average of 15 years of general practice experience. Participating doctors worked in CHCs located in eight of Shenzhen’s 10 districts (see Fig. 1), with 10 doctors from clinics based in coastal districts compared to seven from inland districts. Apart from two doctors, all worked in different clinics. According to information provided by the participants, clinic size varied widely from 2 to 26 GPs and served a residential population ranging from 2000 to 50,000. On average, inland clinics served larger residential communities (Inland: 27,666 versus Coastal: 18,864 residents) but occupied smaller premises (average floor size Inland: 1276 m^2^ versus Coastal: 1314 m^2^) and had similar-sized healthcare teams (5–6 doctors and 5–6 nurses) to coastal clinics. 14 doctors reported having one or more Traditional Chinese Medicine (TCM) doctors working full-time in their CHC.

The following section presents the key findings of this study. More in-depth presentation of the responses in Mandarin and in English are presented in Additional file 2.

### Current practice (TDF Domains 1–5)

#### Awareness of depressive disorder, prevalence and symptom profile

Overall, doctors estimated the clinic-based prevalence of depressive disorder to be below 2% (range 0–40%). Several doctors explained, that whilst they were aware of the condition, it was not their general practice to diagnose it (Table 1: Knowledge 1.1).

**Table 1:** The theoretical domains and key findings

*1. Knowledge*
1.1. Doctors are depression aware but do not actively diagnose
1.2. Patients present with somatic symptoms of depression and do not talk about their feelings
1.3. Key motivations for consultation are insomnia and desire for a “leave-from-work certificate”
*2. Optimism*
2.1. Doctors perceive a sizable mental health treatment gap
*3. Beliefs about consequences*
3.1. Depression is not considered to be a treatment priority in CHC
*4. Memory, attention and decision processes*
4.1. No standardized guidelines for the management of depression at CHC
4.2. Two systems share responsibility for depression care: CHC are focused on initial assessments, general counselling and patient education; Hospital is focussed on diagnosis and treatment
4.3. Traditional Chinese Medicine plays a role in depression treatment
*5. Skills*
5.1. Limited awareness and use of depression scales/screeners by CHC doctors
5.2. Doctors are generally pessimistic about screener utility and effectiveness
5.3. Doctors actively choose time appropriate tools to support diagnosis
*6. Beliefs about capabilities*
6.1. Doctors receive limited professional development
6.2. Doctors’ confidence in their ability to treat is low
*7. Social/professional role and identity*
7.1. Doctors are not psychiatrists
7.2. Doctors protect patients from stigma by avoiding a depression diagnosis
*8. Emotion*
8.1. Doctors fear making treatment mistakes
8.2. Doctors are not attuned to providing psychotherapy
*9. Environmental context and resources*
9.1. High volume of patients and short consultation times at CHC
9.2. Limited trained mental health resource at CHC level
9.3. Limited trained mental health resource at hospital level
9.4. Patients lost to a developing referral system
9.5. Poor CHC ability to follow-up patients
9.6. No anti-depressants at CHC
9.7. Doctors without access to anti-depressants are un-empowered to treat
9.8. No private space/designated consultation room for mental health conditions
*10. Social influences*
10.1. Poor general/community health literacy
10.2. Chinese underlying culture: loss of face accentuates poor health seeking
10.3. Intense stigma associated with the main speciality hospital
10.4. Community induced isolation and discrimination
10.5. Family members are important facilitators for patient care
10.6. Poor family understanding of depression can lead to poor treatment outcomes
10.7. Poor employer attitudes towards depression
10.8. A climate of poor public-opinion and trust in the medical profession
*11. Behavioral regulation*
11.1. Require depression-specific policies for patient reimbursement
11.2. Require doctor incentivisation (like psychosis polices)
11.3. “One psychiatric doctor per community health centre” facilitates passing down and cross-referral
11.4. Establishment of dedicated mental health department at local hospitals
11.5. Review of “five in one policy”
11.6. Stronger health promotion on world mental health day
11.7. Use of e-health to vitalize resource and reach more patients
*12. Reinforcement*
12.1. Improved doctor training with special instruction in mental health
12.2. Access to Western Medicine and improved consulting environment
12.3. Improved mental health literacy
*13. Intentions and 14. Goals*
13.1. The care of depression patients to be more strongly prioritised
13.2. Good psychological health is an important component for quality of life
13.3. Timely management of depression prevents suicide

Most community health service centres rarely diagnose depression, and our centre doesn’t diagnose it basically. We won’t diagnose the patient as depression even though we suspect it. (D14)

Upon reflection of the patient profile, all doctors admitted that they regularly saw patients with “depression symptoms” or “patients with depression tendency”, thus framing a context-specific language (lexicon) from the research outset. Nearly all doctors perceived the presentation of depression to be different in China in comparison to Western countries. In particular, patients avoid discussing their feelings and can even appear “non-reflective” (Table 1: Knowledge 1.2). Some doctors elaborated that doctors are viewed as strangers, who’s role is to pass patients onto the specialist thus deep privacy barriers exist. Instead, patients widely present with general, all-over, body discomfort saying that “they feel in a bad mood” or “they don’t feel quite right”. Several doctors identified insomnia, work worries and the desire to have a “leave from work” certificate as the chief motivation for consultation (Table 1, Knowledge 1.3).

He won’t talk too much about the internal reasons. He will only tell the ones who he trusts; but to us he may feel (the doctor) is an outsider and he may not talk so deeply. (D01)

Patients come to see us directly not because of mental reason. They come to see us due to discomfort such as insomnia, fatigue and so on. (D07)

#### Perceived treatment gap for depressive disorder

Doctors were asked about how optimistic they were that patients with depression symptoms would receive appropriate care for their condition (Table 1, Optimism 2.1). Doctors were not optimistic that patients with identified depression symptoms would receive appropriate care with the majority of doctors estimating that less than half of these patients would follow-through with their referral to see a specialist. Several doctors expressed a sense of hopelessness when talking about the potentially high numbers of patients with depression and the limited resources allocated to mental health. By acknowledging higher rates of depression in the community, they feared they would be overrun with patients with no way to treat them. (Potential barriers to the receipt of care are explored in later sections.)

We have very few psychologists with certifications. If general practitioners screen all these depression patients out, what should we do? I know that 20% people have depression, and the proportion of anxiety disorder is also very high. The reality is, where should we refer these patients to? As you see, there are 20 million people in Shenzhen and there is only one Kangning Hospital with only a dozen of physicians inside. You can calculate the number, 20% of 20 million. How should physicians deal with such a huge number of depression patients? (D09)

#### Status of depression treatment in CHC

Doctors were asked to reflect as to why depression patients may not receive care (Table 1: Beliefs about consequences 3.1). It was perceived that most CHCs do not consider depression as a priority condition for treatment. This is consistent with their perception that treatment falls into the domain of specialists (see TDF Domain 4 and Domain 7). Overall, doctors believed it was a matter of fairness to treat all patients equally. As a non-acute condition, it is acceptable for depression patients to take-their-turn alongside other patients in the queue to see the doctor with prioritization (according to clinic protocols), only occurring in the event of an emergency (e.g. threat of suicide). Doctors worried that prioritizing depression patients adversely identified them, compounding stigma and making it harder for depression patients to return to the clinic for care.

There shouldn’t be any priority, since we treat all patients consistently. …. It can’t be that the patient who has mental problem comes and I’ll just need to give him special care. There is no such thing. (D06)

#### Doctors current approach to depression care

**Fig. 2:**
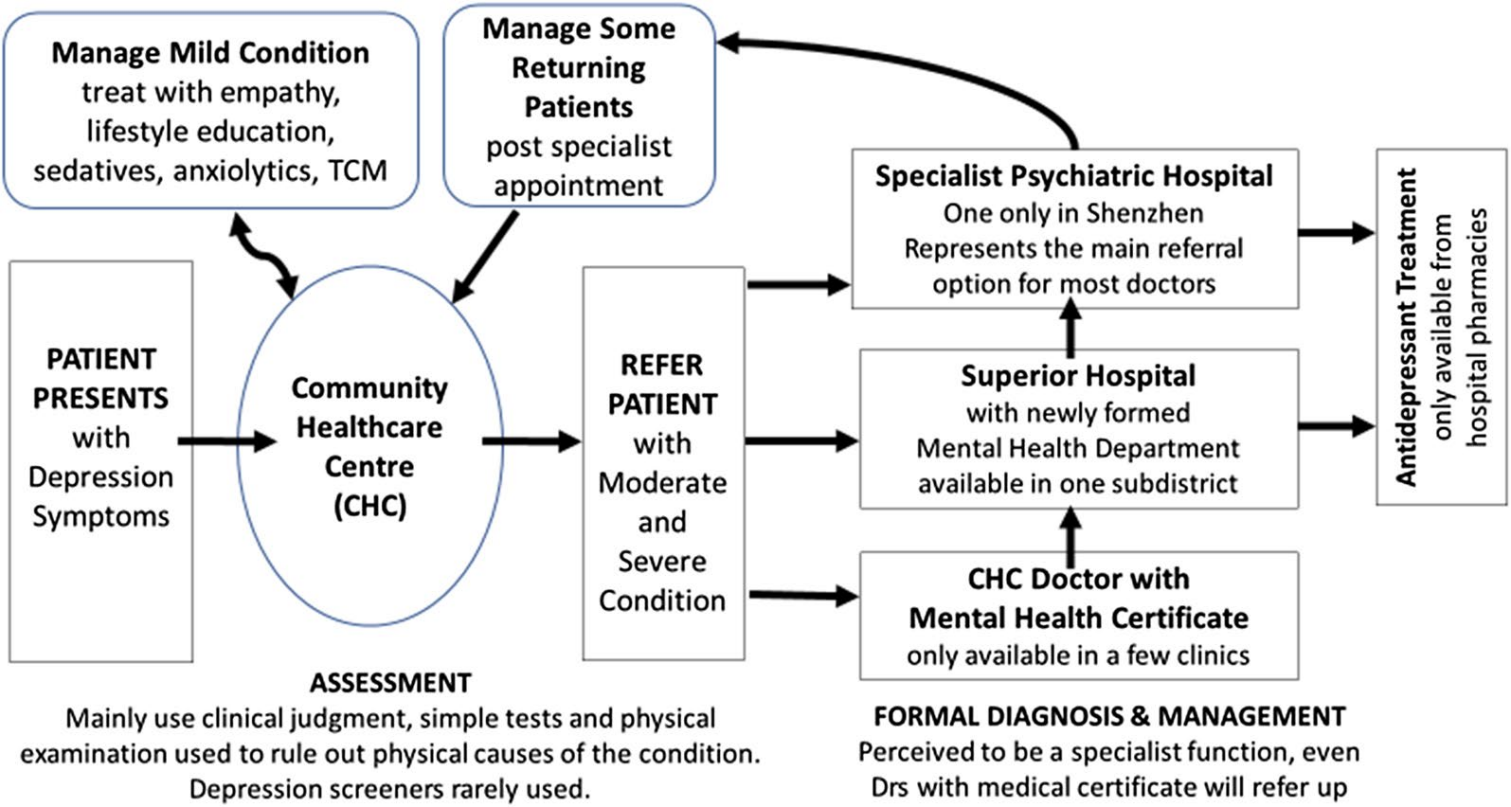
Patient pathway and doctor decision-points for depression treatment and care

Doctors described the key assessment, treatment and follow-up decisions they currently make when faced with a potential depression patient (Fig. 2). CHC doctors, currently play a limited role in the diagnosis and treatment of depression (potential reasons for this limited role are discussed in later sections). Their role is to conduct physical examinations, request laboratory tests and rule-out physical causes of disease before referring patients either to the in-house doctor with a specialist mental health certificate (if available) or most commonly, directly to the only public psychiatric hospital, Kangning, for diagnosis and treatment (Table 1: Memory, attention and decision processes 4.1). Antidepressant treatments are only available from hospitals. In one catchment area, there is the option to also refer to the new mental health department of a local hospital.

The majority of doctors provide limited on-site care. Initially, they give empathy and comfort before providing basic counseling and lifestyle education. These longer consultations take place in the clinic by arrangement of the doctor, who asks the patient to return when the clinic is less busy.

We spend much time communicating with patients to let them understand that depression is a common disease, which is just like cold. Besides, we need to let them know that some diseases can be cured via life style improvement, individual adjustment or psychological guidance. (D08)

Additionally, they will treat insomnia with either Western (e.g. Diazepam, Estazolam) or Traditional Chinese Medicine (TCM), or both. Even though most doctors recommend TCM (e.g. acupuncture, cupping, herbal brews), over half prefer Western medicine (obtained from the hospital) as first-line treatment. However, should patients specifically seek TCM, they would honor this with many doctors having been trained in TCM as part of their medical degree and TCM specialists available at most CHCs (Table 1: Memory, attention and decision processes 4.3). Most doctors indicated there wasn’t any other psychological support services in the community, instead key services are hospital-based.

If they come to see Traditional Chinese physicians on purpose, definitely, we prescribe Chinese medicine to them……In fact, there is acupuncture therapy for insomnia and headache, and we prescribe traditional Chinese medicine in combination with these therapies. (D16)

#### Current usage of mental health guidelines

Most doctors were both unaware of and don’t use any guidelines for the treatment of depression patients in primary care (Table 1: Memory, attention and decision processes 4.1). Diagnosis was generally up to their own clinical ability, in most cases, developed through practical experience and in a few cases, through self-directed reading of medical journals.

We assess according to physician’s clinical experience. Therefore, community health service centers are not professional in terms of mental disease diagnosis currently. (D13)

Doctors tended to separate out their role from that of a psychologist (or doctor with a special certificate in psychology) or policy-maker whom they thought would be more aware of guidelines. Some doctors indicated too, that implementing mental health guidelines just wasn’t in their clinic’s consciousness (see Domain 7 for more information on doctor role).

#### Current usage of assessment tools

It is not common practice for CHC doctors to use screening instruments during consultations, although many doctors ask screening type questions casually to help them with their diagnosis (Table 1: Skills 5.1). These instruments are considered as a specialist tool, are not readily available as a CHC resource and many doctors are unfamiliar with their specific names. In addition, there was the prevailing feeling that they were considered unacceptable by patients and doctors due to reasons of stigma (see Domains 9 and 10 for further explanation). A few doctors, however, download questionnaires on their mobile phones when needed. Amongst the minority of doctors who regularly use screening instruments, the Kessler Psychological Distress Scale (K10) and Patient Health Questionnaire 2 and 9 (PHQ-2, PHQ-9) were mentioned.

It’s not proper to take the scale to ask patients questions directly because they may feel disgusted. Basically, we talk with patients with some communication skills, not just asking questions rigidly. (D02)

Doctors were pessimistic about the utility and effectiveness of screening tools (Table 1: Skills 5.2). They regularly mentioned that time constraints, both on the part of the doctor and the patient, rendered many screeners impractical to use. Doctors feared lengthy screeners would drive patients away. One doctor, experienced with using scales, expressed his concern that patients could manipulate the screening outcome.

However, we feel that the scale can’t represent the reality, as our patients are very smart, and they know what the scale is for… for instance, when they wish physicians to feel the severity of their disease, they tick the serious options; when they want to avoid negative outcomes, they select mild options. In a word, they tick answers according to their subjective wishes, rather than their real status. In later psychological consulting, physicians find that their status is not so severe as or more severe than the scale outcome. (D14)

In a few clinics where screening is an option, doctors counter short-consultation times by choosing time-efficient screening tools (e.g. PHQ-2 or bespoke questionnaires) (Table 1: Skills 5.3). These provide doctors with an initial indication of depression and a reason to ask patients back for further investigation using longer screening instruments at a time when they are not so busy.

…that’s why we use PHQ-2 very often. There are problems if the score of PHQ-2 is higher than 3, so we use PHQ-9 to make another evaluation…. As for mild patients, we ask them to visit us for several times when there are not many patients, as we have to control the counselling time for every patient when we are busy. (D10)

### Doctors’ psychological response to providing depression care (TDF Domains 6–8)

#### Doctors personal beliefs about their capabilities

Many doctors acknowledge that they have received limited training for community-based mental health care and express deep concerns about their levels of competency, from their ability to diagnose (see Domain 5, Skills) to making medication-choices based on side-effect profiles, and how to communicate the diagnosis (Table 1: Beliefs about capabilities 6.1–6.2). This is exacerbated by a time-poor, work-culture, devoid of case-sharing within CHC and between CHC doctors and specialists. Nobody had access to a formal peer-based mentoring scheme and only a few doctors worked in clinics where visiting specialists provided regular mental health training sessions to support ongoing professional development. In the absence of learning-support structures, most doctors, confide that their confidence is low and that the treatment of depression patients is often considered beyond their abilities.

With respect to treatment… training about it is rare. Physicians are not so confident in treating patients like this, and don’t know what therapies are proper for the patients. (D11)

#### Doctors beliefs about their professional role and identify

Doctors were asked to reflect on their role with regards to assessing and managing depression. Despite increasing policy-pressure to take on primary responsibility for treating mental disorders in primary care, most doctors, even those who hold a special mental health certificate, appear resistant to diagnosing depression. Doctors clearly differentiated their skill-set from that of psychologists/psychiatrists and many do not believe it is their place to provide the diagnosis (Table 1: Social/professional role and identity 7.1). Given the insufficient time they have to asses for depression, they prefer to refer suspected cases to the superior hospital in their district where there is ready access to drug treatment (unlike CHCs) and a formal diagnosis can be made (see Domain 9 Environmental Health System Resource for further explanation).

I don’t want to first make this diagnosis, because after all I’m not a psychiatrist…Although I have the psychological counsellor certificate, in our country it requires…Class Two Psychological Counsellor in order to prescribe this medicine. (D03)

Doctors are highly aware of depression-related stigma in the community. Many believe an important element of their role is to maintain patient privacy and to avoid stigmatizing patients with a depression diagnosis (Table 1: Social/professional role and identity 7.2). They use a range of strategies to minimize patient confrontation and manage prejudice against the condition. For example, they might ask the patient first, what they think they have, or if they want to self-test with a questionnaire. They rarely tell the patient that they have depression, instead, they use euphemisms or discuss depression through the conduit of neurasthenia or sleep disorders. Some doctors also actively bypass referring patients to Kangning (choosing to refer to superior hospitals instead) to avoid stigmatizing their patients. Overall, doctors were clear that they needed to manage patient perceptions of depression to facilitate treatment adherence.

We are cautious for the diagnosis because it’s a taboo for some people, and they may feel embarrassed. We also don’t say it out easily when suspecting that it’s depression. Perhaps we’ll tell the patient euphemistically. Maybe I’ll tell patients that their pressure recently is somewhat great, and it will be possible for them to get depression if they don’t pay attention to it…..We don’t dare to recommend the specialized hospital dedicated to mental diseases, but recommend general hospitals because there is psychological department in large general hospitals. (D13)

#### Doctors underlying emotional influences

Doctors were asked to consider any personally significant factors that might shape doctor behavior in relation to the care of depression patients. Doctors were candid with their responses with several revealing how they experienced low levels of self-esteem and confidence in their medical abilities. The long course of disease for depression was perceived to be particularly hard to manage placing doctors under immense pressure. One doctor described how doctors even begin to fear patients with complicated case histories and worry about making treatment-mistakes (Table 1: Emotion 8.1).

Doctors are also very afraid of patients who cause accidents! Like those mental health patients…(who) sometimes will fall ill, or don’t take medicine in time….and cause accidents at the slightest stimulation. It will be very troublesome!.. Many doctors…..are not specialized in this, and are not very familiar with this area … so they feel the pressure is big. (D05)

Another doctor queried their own professional robustness with delivering non-drug interventions such as psychotherapy. For reasons of self-preservation, the doctor admitted that the confronting nature of the risk factors for depression was a deterrent (Table 1: Emotion 8.2).

There is also a bit of personal factor which is I am not willing to develop in this direction…because the truth they reveal must be sometimes torture, domestic violence and other types of unpleasant things… If I come across too much of this kind of negative darkness…. I think my emotions will also be affected. So, I don’t want to be taken into the darkness by them. (D03)

### External influences (Domains 9–10)

#### Health system and immediate work environment context

The most widely-cited health system barrier to treatment success, as mentioned by most doctors, was the high volume of patients regularly attending clinics resulting in very restricted consultation times (Table 1: Environmental context and resources 9.1). This factor alone curtails doctors’ ability to initiate appropriate screening and exacerbates poor case detection.

Firstly, there is deficiency in the aspect of depression finding, and perhaps patients with depression might be omitted. Commonly, we have too many patients, and perhaps we need to treat a patient every 2–3 min. We have no time to ask his medical history at all, and it’s also impossible to discern whether the patient has psychological problems or not. (D12)

For patients identified with depression, doctors are then faced with a resource dilemma. With limited personnel qualified to conduct psychotherapy and antidepressants standardly unavailable at CHC, their only option is to refer onto the small pool of hospital specialists and in the process, risk losing these patients in the over-burdened hospital system (Table 1: Environmental context and resources 9.2–9.3).

Only a few physicians in community hospitals are able to deal with depression…..Most physicians don’t know how to diagnose depression or when to screen. (D10)

The psychological doctors….are very few…(but) the patients are packed over several floors. (D04)

Nearly all doctors related experiences where many of their patients, especially those with milder symptoms, do not receive care. Collectively, they identified weaknesses in the referral process as follows: Firstly, doctors avoid marking patient’s health record with a depression diagnosis. Secondly, patients often avoid specialist treatment. Thirdly, the current electronic referral system only records a confirmed diagnosis. It neither tracks patients’ progress to specialist care nor details consultation outcomes. This situation is further hampered by patients’ lack of willingness to provide accurate contact details making telephone follow-up calls, post initial consultation, ineffective (see Domain 10 social influences for further explanation) (Table 1: Environmental context and resources 9.4–9.5).

When we feel that their status is somewhat severe, we refer them to hospitals, and we seldom trace them after the referral. If the referred hospital has confirmed the diagnosis of depression, patient information would be sent back to the community healthcare centre… Only under this circumstance would we trace them and follow them up. If their severity hasn’t met the diagnosis criteria, we don’t follow them up. (D17)

Currently, antidepressants are either excluded from clinic formularies or have restricted access (Table 1: Environmental context and resources 9.6). Many doctors emphasized that having limited or no access to appropriate drug treatment at clinic level was a key barrier that must be addressed in order to improve patient treatment conditions. Apart from preventing the doctor from providing timely treatment, one doctor explained that by having treatment control in the hands of the hospital, the doctor’s ability to develop a relationship with the patient was curtailed (Table 1: Environmental context and resources 9.7).

Currently, common drugs for anti-depression are managed as antipsychotics. Many leaders think community health service center should not have this kind of drugs. However, what our centre requires the most is these drugs. (D08)

Community healthcare center has no diagnosis capability and no corresponding drugs for them. Some patients haven’t even met the physicians here, so there is no face-to-face interaction. They get drugs at Kangning… therefore, some of them are unwilling to accept our management, as they feel it unnecessary. (D16)

Most doctors had either previously or currently experienced conducting consultations in a highly pressurized environment, devoid of privacy and space. Very few doctors had ready access to a consultation room specifically dedicated to mental health consultations. In the older-style clinics, the open-plan consultation rooms negate privacy for all routine conditions including mental health complaints. Even in the newer clinics, poor sound-proofing between rooms makes privacy hard to guarantee as patients outside can hear the full nature of the consultation discussion. In many clinics, doctors strive to ask patients to return at quieter times when they can book-out a room. However, this ongoing struggle to find private space can curtail doctors’ best intentions and they end-up seeing only those most in need (Table 1: Environmental context and resources 9.8).

However, the situation is rare because we have so many patients to see, and we need to arrange a room for them separately, and thus we only select one intractable patient from them. (D17)

#### Social influences

Doctors were asked to consider the influence of society, the wider community, China’s general work culture and patients ‘attitude on doctors’ ability to initiate depression care. Many doctors explained that they worked in an environment where the general health literacy and understanding of mental health in the community was low (Table 1: Social influences 10.1). Many patients are neither aware of the symptoms of depression, nor that it is treatable. Depression is widely confused with psychiatric disease. Thus, in an environment where patients are slow to discuss depression even with their friends, doctors are even more unlikely to be consulted.

In Chinese society, common people consider psychological problems as psychiatric problems or mental illnesses. They don’t accept it! Moreover …. we don’t have many websites, newspaper, or free calls for consultation, so they don’t talk with others about their problem, which worsens the illness. (D09)

The cultural-specific concept of “loss-of-face” was thought to be deeply rooted in patients’ consciousness and detrimental to the early detection of depression (Table 1: Social influences 10.2). This “innate” fear of exposing their personal regrets, weaknesses or relationship failures (including being victims of violence) coupled with a limited understanding of mental health inhibits them from consulting their local doctor. Several doctors mentioned a cultural predisposition to self-regulate illness and delay health seeking with the consequence that doctors may only see patients later in disease progression when specialist attention is critical.

Chinese people are afraid of stigma!… Chinese people are like, let the household disgrace be buried inside the house. Usually they keep it to themselves. (D04)

Stigma surrounding Kangning, the only public psychiatric hospital in Shenzhen acts as a significant treatment barrier (Table 1: Social influences 10.3). Several doctors related how patients simply refuse to attend their referral appointments at Kangning. Amongst those patients that eventually do consent to specialist attention, the fear of stigma is so acute that patients have been known to deliberately misinform the hospital administration of their personal identity. The futility of this situation acts like a negative reinforcement loop with doctors then avoiding identifying and referring patients for treatment (see Domain 3 beliefs about consequences and Domain 7 social/professional role and identity).

When we want to refer (patients) to (specialist) hospitals, they don’t go there, as in Chinese culture, it’s a taboo to see psychiatrists or psychologists, as most people are afraid of being considered as a psycho. (D03)

Stigma in the community, in the form of idle gossip, can lead to patients becoming increasingly isolated at times when support is most needed (Table 1: Social influences 10.4). One doctor relates, how even a home visit by medical personnel or other services involved in community care (including the police) can compromise a patient’s wellbeing. Furthermore, patients, without family protection, are likely to become increasingly alienated.

When we go to the patient’s home for follow-up, nearby residents may pop their head out to see what happened and gossip because there are many people including policeman.^3^ They think the person we are visiting has committed a crime, which then increases the mental stress of the patient. (D12)

With regards to the general workplace culture in China, employers rarely accept, or support employees with depression (Table 1: Social influences 10.7). For instance, one doctor explained that to attend any medical appointment, employees must ask for permission to leave their post, as time away from work directly impacts company productivity. Secondly, managers can be dismissive of symptoms of depression and it is easier to obtain permission for more recognizable conditions (e.g. fever). It was perceived that with the threat of redundancy high, employees are unlikely to discuss their concerns with work colleagues and even in severe cases employees seek treatment out of work-time.

When you ask your employer for leave, he/she may ask you your problem. The employer may approve it rapidly if you say you have a fever or something else. However, if you tell him/her that you have had poor sleep or bad mood, he/she may advise you not to think too much and work harder. (D02)

Despite low awareness of depression, work-anxiety is more widely recognised although it is generally viewed as a positive attribute, in keeping with a good work ethic.

I think they, because of the anxiety, should perform even better at work, such as complete tasks very quickly …… it hasn’t affected his work. So people around him might just feel he is a bit anxious, and there doesn’t exist that kind of (suspicion that the patient has mental problems). (D03)

Most doctors commented that the family (and friends) play a vital role in ensuring a patient receives care and support (Table 1: Social influences 10.5). Additionally, the family: is often the first to be aware of behavior changes; is instrumental in getting the patient to the initial consultation; plays a key role in monitoring treatment; and ensures the patient attends hospital appointments.

If family members are all very positive, there is no problem. They encourage patients to see physicians because of anxiety or depression, as they feel that problems can be solved after taking medicine. If family members consider it as a scandal and keep it in secret, the status would worsen. (D09)

Conversely, patients living in households with a poor understanding of depression and high levels of stigma were less likely to receive appropriate care and their condition can worsen (Table 1: Social influences 10.6). Many doctors described homes where a general feeling of hopelessness prevails, and patients are discriminated against by members of their own family. Guardians repudiate the patient’s condition and refuse doctors access to these patients. Many doctors, when making their follow-up calls had experienced family members putting the phone down or even verbally abusing them.

The family will say, don’t call me! I don’t have this (family member) with mental problems at home. He also feels if he has a family member who has such mental problems, this will be a very shameful thing. (D01)

Poor public opinion concerning the medical profession directly hampers their ability to effectively manage and treat their patients (Table 1: Social influences 10.8). Most doctors related experiences of patients rejecting their advice: declining depression screening; avoiding hospital consultations; declining treatment; and ignoring follow-up phone-calls. When speculating on potential reasons for this rejection, many doctors concluded that poor trust in doctors was the major obstacle.

Currently, the relationship between doctors and patients is somewhat tense. The public opinion makes people think it requires a high cost for getting medical treatment, and all the money is earned by doctors. (D07)

If the patient is positive, his/her compliance is high and he/she trusts me, it will be quite helpful, and the disease can be cured quicker relatively. If the patient doesn’t trust me or he/she doesn’t understand the disease well, or he/she rejects it subconsciously, I will have no way to treat it. This is the obstacle. (R08)

### Doctor identified enablers and needs for improved depression care (Domains 11–12)

#### Health system policy enablers

In general, doctors were not overtly aware of specific mental health policies, although collectively, doctors highlighted a range of initiatives that affected their day-to-day work both positively and negatively. Doctors thought constructively about what approaches would enable them to improve depression care for their patients.

The most widely cited concern, mentioned by over half of the doctors, was that current health policy was directed at severe mental disorders, not at depression. In particular, the medical insurance system does not reimburse drug treatment for depressive disorders nor are there incentives for doctors to diagnose milder conditions (Table 1: Behavioral regulation 11.1). In contrast, doctors referring patients to Kanging hospital, with a correct psychiatric diagnosis (e.g. schizophrenia) are eligible for a financial reward. Thus, many doctors hoped that similar models of reimbursement and incentivization could be applied to depression management.

Now we pay much attention to severe mental disorder, so there is corresponding policy support, and drugs for mental disorder are free after patients apply. However, there is no such policy for depression. (D10)

If the GP discovers one of these cases (schizophrenia), there will be certain money (reward) to encourage you to discover and report (more cases). (D04)

Some doctors were aware of the “One Psychiatric Doctor per Community Health Centre” directive to improve mental health care in CHCs. These doctors were optimistic about this policy with one doctor noting improved referral efficiencies (e.g. down-referral of patients from hospital to CHC) whilst another doctor highlighted the cross-referral benefits of having a locally-based expert to deal with complex cases.

If the patient has already a confirmed diagnosis, … the diagnosed patient will be recorded in the system (by the hospital), then…our doctor in charge of psychiatric prevention (in the community centre) can also see this patient….We call that passing down to the doctor who is in charge of mental health. (D06)

Some doctors in our centre received training about psychological counselling before. For instance, if the patient is not familiar to me, and I’m not good at the treatment of the disease, I may ask my colleague for consultation …..(D02)

Several doctors had considered applying for the government-assisted training scheme to prepare for the yearly examinations for this additional certification. However, they also mentioned that with heavy workloads and many clinics short-staffed, this would not feasible for all doctors.

One doctor explained how a recent merger of several hospitals, (including the People’s Hospital, Traditional Chinese Medicine Hospital, and Maternal and Child Health Hospital) into the new Luohu Hospital Group, had facilitated the establishment of a dedicated mental health department. This initiative provides both an important referral alternative to Kangning Hospital, bypasses concerns of stigma (see Domain 10 social influences) and has opened-up communication channels between doctors and specialists for the ongoing follow-up of patient cases. The specialist in charge also has taken on the responsibility of running regular training sessions in community clinics.

Now (the Group) has established a mental health department …..as well as some Wechat communication groups… When we come across some patients and do not know how to deal with it by ourselves, we can consult that Director in that chat group…. If we can’t solve it ourselves, we might …..make a referral up (to the Group), we can still have certain communication with the doctor who accepts the referral. (If) you make a referral to Kangning Hospital, you will have a hard time track that patient’s progress. (D05)

Patient’s insecurity with regards to doctors’ trust, is strongly connected with issues of patient confidentiality and how the current policy practice for patients with psychiatric disorders (not depressive disorder) undermines this trust. Doctors must, according to “five-in-one policy”, provide the partner organizations (i.e. hospital, community case management, neighborhood committees, schools and police^4^) with the details of high-risk mental health patients to facilitate multi-sectorial care. Patients with little overall understanding of mental health and unaware of the differences between depression and psychosis assume that doctors will need to inform the police of their condition. One doctor queries whether the emphasis of this policy is misplaced, in that it is more focused on protecting community members than looking after the patient. In following it, patients can potentially lose their jobs and homes. Another doctor relates that they go to extreme measures of giving gifts to encourage patients to attend the physical examinations (related to depression case determination) to overcome patients’ fears about privacy.

Why the policy of “five-in-one” is necessary? Firstly, physicians feel unsafe. Secondly, patients reject it themselves. They want to protect their privacy and don’t want others know that they have the disease. (D12)

A few doctors were aware of general health care promotions centred around “World Mental Health Day” and initiatives for depression screening in key target populations, which were seen as precursors to policies in the future.

Shenzhen has already been a leader in this field, as this city has established many policies, including screening children with autism, maternal depression and elderly depression. However, it’s the preparatory work of a program, and it hasn’t been conducted in clinical practice. (D09)

One doctor pointed out that to fill the void of poor psychological services in the community, internet counselling was increasingly being sought and provided outside the official health system. The doctor highlighted that it was a viable career opportunity for medical personnel who were thinking of retiring.

I have a friend, a nurse, she also holds the psychological counselling certificate, (and) she is an online psychological counsellor. She is paid online by virtual money…..Because ….her career is reaching its end and she doesn’t want to end up with no economic back up …. she’s thinking…..she could change career to become a psychological counsellor. So she’s doing that online. (D03)

#### Doctor identified needs for improvement to depression care

Practical mental health training was most widely cited, by the majority of doctors, as the priority need to overcome the challenges in this area. Training needs to be specific to general practice, practical in nature and encompass both diagnosis and treatment options.

Firstly, some relevant training needs to be provided. Besides theoretical training, we also need role-play for better understanding about it. Actually, we learned relevant knowledge before, but it was extremely shallow. It will be the best for us if there is practical training. The integration of theory with practice is important. (D07)

The majority of doctors reported that their clinics didn’t have access to private consulting rooms (or space) or sufficient time in which to conduct mental health assessments or general counselling sessions. Many doctors also highlighted having access to appropriate drug treatment as a priority need.

It is the best if we can prescribe corresponding drugs, have enough time and a quiet consulting room. It’s impossible to work well with a lot of patients in the consulting room. (D17)

Doctors articulated that mental health gap could not be resolved by improved doctor skills and health system reform alone. Most doctors indicated that until there is a societal change in attitudes towards depression their jobs would remain difficult. Many advocated that government should invest in raising the health literacy of the nation, in particular, by improving both access to and quality of information available in the public domain including internet search engines (e.g. Baidu). Improved awareness was seen to benefit treatment outcomes and even bring an end to discrimination.

If the patient is aware of his illness, he can only have a search in Baidu. But much of the information in Baidu^5^ is useless. Trash information! There is no… professional information that could provide useful advice to the patient…or could help people realize that this is in fact a very common issue, and not a very embarrassing problem. I think this is an issue about public awareness of this disease. The key is the awareness. If everyone thinks it’s the same as having a cold or fever, ….. then everyone will be able to treat it normally. You have this, (and) I also have this, (and) there won’t be any discrimination. Hmm, there won’t be any discrimination, then people can be open to speak about such a thing. (D01)

### Doctors’ desire to change (TDF Domains 13 and 14)

Whilst not currently considered a priority condition in most clinics, there was the prevailing opinion that depressive disorder should be more strongly prioritized. People with depression are generally those who are disadvantaged or vulnerable in some way, with poor ability to wait and thus doctors need to be additionally vigilant to help these patients to avoid losing them and postponing diagnosis.

I think there should be a health priority. Patients like this get easily annoyed if they have to wait for the diagnosis for a long time or their attitudes towards physicians are not good or they are not well cared for. …. Perhaps it’s acceptable for general patients to wait for a long time, but patients with emotional disorder can’t wait for such a long time and then they may go away, which will cause the delay of disease. (D13)

A minority of doctors indicated that depression was already a priority in their clinic. These doctors were aware that the prevalence of depression was increasing in their country and expressed the view that if you do not look after patients’ psychological health you will have poor outcomes for all conditions and can result in suicide.

In my perspective, we need to pay attention to patients’ psychological health, no matter whether they suffer anxiety or depression or not. We need to intervene when discovering such cases. I pay more attention to patients’ psychological health, as good psychological health is good for their quality of life. (D15)

Depression is a hidden disease and it will cause bad consequences if it’s not solved timely, and even cause tragedy. (D11)

## Discussion

This research brings new insights about how primary care doctors in Shenzhen perceive their role within the health system and their beliefs and concerns about their own professional capabilities. Despite national healthcare reform [39] and global healthcare trends which advocate the integration of mental health care into primary care [40], primary care doctors perceive the formal diagnosis and treatment of depression to be the domain of hospital-based specialists. Even though they are aware of a mental health treatment gap in Shenzhen and the potential benefits of early depression care on community well-being, they remain hesitant to “formally” diagnose depression and may inadvertently delay appropriate treatment. They acknowledge, however, that the practice of referring patients to specialists often masks their own low treatment confidence and protects them from causing “accidents”. Furthermore, they described how community health centres (CHC) do not have any specific treatment protocols (e.g. international, national or clinic-specific guidelines for the assessment, management and follow-up of depression), nor standard access to depression screeners, nor an established educational network (e.g. regular case-sharing and mentorship with other peers and mental health specialists) to support them with their assessment and management decisions.

Consistent with China’s health policy [39], doctors emphasized their urgent need for mental health training, specifically designed for general practice (not hospital specialists). Ideally, professional development programs would define the scope and role doctors have for improving depression care within their local health care system; teach relevant diagnostic skills; strengthen treatment confidence; provide guidelines for ongoing care and develop a multi-disciplinary mental health support network (local and international). Recent training initiatives with primary care doctors in Shenzhen have demonstrated how experiential teaching approaches can effectively deliver targeted and practical training solutions [51].

The World Health Organisation’s Mental Health Gap Intervention Guide (mhGAP-IG.v2) [52–57] is a decision-support tool for non-specialists to assess, manage and follow-up a range of MNS disorders (including depression) using evidence-based and financially affordable treatment options [58, 59]. It has already been successfully used for mental health scale-up in 90 countries [54] and learnings from recent contextualization research highlights its potential for China to use as a framework to review mental health policy, as a diagnostic-aid at the point-of-care or as a training resource. Furthermore, an adapted guide, which takes account of Shenzhen’s specific sociocultural factors that influence illness onset, presentation, health-seeking behavior and health-system response to depression [60], may provide these community health centres with a training template to develop their human resource.

Primary care medical leaders in Shenzhen are both willing and increasingly in a position to make real improvements to providing appropriate care to depression patients, especially when supported by the active health system reform. Similar to other studies [39, 61, 62], high patient-loads, short consultation-times, limited patient privacy and inexperienced staff were seen by doctors to hinder the initial assessment and identification of depression. Doctors welcomed the governments’ response to upskilling primary care resource through the “One Psychiatric Doctor per Community Health Centre”, a recognized goal of the 2015–2020 National Mental Health Work Plan [38]. However, doctors agreed that they needed greater treatment autonomy through the inclusion of antidepressants on the essential medicine’s list for CHC. This change alone, would significantly elevate the role of the CHC to a key treatment hub for depression and allow doctors to initiate and take control of their patients’ treatment plan in-line with the recent Lancet Commission [40] on global mental health.

At the funding level, doctors look to recent changes in reimbursement policy for psychosis drugs and are hopeful that similar advances can be made for depression so that patients can have more ready access to drug treatment.

Additionally, doctors require an improved management referral system. The current model which does not track patient outcomes, limits doctors’ ability to check patient compliance and provide follow-up care. It effectively renders patients “lost from the healthcare system”. These findings are supported by National Survey data (2017) which estimates that over half of community health centers still do not have an electronic medical record system in place and of those that do, only 40% can link their systems with the hospital to facilitate patient referral. Current systems have been developed by multiple IT providers without a centralized plan or uniform protocols and data linkage is both poor and hard to interpret [39].

Doctors are also searching for alternative referral options. Locally-based initiatives to establish dedicated mental health departments at local hospital level were well received as they provided doctors with a socially acceptable option for higher level care (i.e. not the only public psychiatric hospital). When looking to increase access to psychosocial care, Shenzhen with its high-tech culture provides a unique site for the development of e-mental health and web-based care. This research draws attention to the feasibility of nursing staff with a psychological counseling certificate providing psychosocial interventions by teleconference. Recent investigations focus on determining the effectiveness of mental health teleconferences in high-income countries [63, 64] with web-based depression screening programs increasingly being tested for use in general practice [65]. Digital technologies overall, offer potential to reduce the mental health treatment gap in low-income countries [66] including internet-based tools to provide cognitive behavioral therapy [67] and assess suicide risk in China [67, 68].

Consistent with other China-specific research [16–18, 32, 69, 70], these doctors suggest that mental health stigma plays a pivotal role in shaping a patient culture of reluctance to seek help for mental health problems, masked symptom presentation and common rejection of specialist help in China. However, this study, describes the high level of stigma associated with seeking help from the only public psychiatric hospital, in Shenzhen. The view of this psychiatric hospital as a place to be avoided at all cost means that doctors attempts to direct their patients to care are undermined. In a climate of poor overall mental health awareness, where few distinguish between different types of mental disorders, public-security focused policies (e.g. the five-in-one policy, which requires doctors to register severe psychosis patients with the police [71]) reinforces stigma by associating any mental health condition with criminality, rather than an illness requiring care. Doctors are fully aware of stigma in society and in their role as “protector” of patient privacy avoid using diagnostic labels. However, by communicating in euphemisms such as “tending towards depression” and not talking openly about the condition, they are inadvertently “keeping the lid on the box”. In accordance with mental health literacy research [72], these doctors reflect that until mental health awareness at the population level is vastly improved, their jobs will remain difficult.

### Limitations and strengths of the study

This qualitative study applied the Theoretical Domains Framework (TDF) from research conceptualization to completion. The TDF was developed by an expert consensus approach to integrate 33 theories of organizational and psychological behavior change into one framework (with fourteen domains), for use by implementation researchers working in healthcare systems and thus was an excellent fit for the needs of this study [43]. Its theoretical origins and breadth of constructs included in domain development, ensured that relevant factors could be systematically identified in Shenzhen’s healthcare context [44]. The prospective use of the TDF can inform future intervention design [73–75] such as the cultural adaptation of the mhGAP-IG to the Shenzhen context and enhance its uptake as a support tool. Most importantly, with its focus on understanding the doctors’ perspective, it informs how behavioral change of healthcare providers can be supported to improve patient care.

This study has been conducted with primary care opinion leaders identified through their participation in the international Monash-Shenzhen Primary Healthcare Leaders Program. Findings from this select group may not be representative of the entire primary care community in Shenzhen. As leaders in their community, however, they may represent progressive views and act as early adopters of new practices. In addition, their international experience allows them to act as a vital bridge between their community and the global discussion on mental health.

This research was not directed at identifying financial barriers and enablers to the management of depression care, which is an important and vital area driving healthcare solutions for the future. Discussions were conducted using an open-ended discussion guide which allowed for the spontaneous reflections of doctors, followed-up with general probes (i.e. What else?). Thus, other than the suggestion that future policy consider drug reimbursement and doctor incentivization similar to psychosis care, this research cannot provide any insight into these drivers.

Interviews were conducted in Mandarin, by a native Mandarin speaking interviewer, whilst the analysis was conducted in English. To ensure that rich contextual examples and cultural nuance were maintained, approximately 10% of transcripts, the key themes, concepts and code frames were back-translated and discussed with interviewers.

## Conclusions and future directions

This research places doctors at the centre of the investigation and by applying the TDF describes how doctors “sense of self” (i.e. personal psychology), organisational and societal barriers shape a standard practice of referral of potential depression patients rather than encouraging community-based symptom management and treatment of depressive disorder. Whilst many health system improvements, such as the inclusion of antidepressant medications in community health centre formularies, the general improvement of the primary care consulting environment and the development of patient referral systems are within reach of current policy directives, widespread mental health stigma continues to be a challenge. Importantly, this study highlights how the provision of appropriate mental health training is central to Primary Care doctors’ professional development, the improvement of their treatment confidence and for the care of their communities.

The findings from this research will inform the cultural adaption of the mhGAP-IG for use by community-based doctors in clinical practice, a follow-on research project being undertaken by our group. Embedding this tool in training programs specifically targeted at primary care would provide doctors with evidence-based guidelines relevant to their context, strengthen confidence and improve capacity to provide assessment and management for depression in China’s primary healthcare sector.

## Additional files

**Additional file 1.** Theoretical domains framework and discussion guide.

**Additional file 2.** Quotes in Mandarin Chinese and English translation.

## Data Availability

Data is stored at the University of Melbourne. The data cannot be freely used, as the study is part of a Ph.D. thesis, with the candidate currently working on the remaining data.

## Abbreviations

MNS: mental, neurological and substance use disorders
MhGAP-Ig.v2: Mental Health Gap Intervention Guide (version 2)
CHC: community healthcare centre
DALYs: disability adjusted life years
TCM: Traditional Chinese Medicine

## Footnotes

1 At the time of research initiation this program was called the Monash-Shenzhen Primary Healthcare Leaders Program. The program has been recently renamed to become: Shenzhen General Practice Clinical Leadership Training.

2 Directed content analysis Hsieh and Shannon [48] identify three types of qualitative content analysis: summative, conventional, and directed. Summative content analysis involves the counting of words or content and the interpretation of that quantification. In conventional content analysis, categories emerge out of the analysis rather than through preconceived categories being imposed on the data. Directed content analysis involves the application of conceptual categories to a new context. Directed content analysis is appropriate to use when “existing theory or prior research about a phenomenon that is incomplete… would benefit from further description,” with the goal “to validate or extend conceptually a theoretical framework or theory” (Hsieh and Shannon [48], p. 1281).

3 The five-in-one policy is directed at patients with psychiatric disorders and potentially violent behavior (not depressive disorder) to improve their multi-sectorial care between services (hospital, community case management, neighborhood committees, schools and police). Thus for difficult cases, the police are often involved in facilitating care. This creates the perception of criminality rather than illness.

4 The five-in-one policy is directed at patients with psychiatric disorders and potentially violent behavior (not depressive disorder) to improve their multi-sectorial care between services (hospital, community case management, neighborhood committees, schools and police). However, it appears that community understanding of mental health is such that depression is potentially confused with psychotic disorders and thus patients worry that doctors will need to inform the police.

5 Baidu is a widely used internet service provider in China.
