# Supplementary material for: Current needs for the improved management of depressive disorder in community healthcare centres, Shenzhen, China: a view from primary care medical leaders
Source: Int J Ment Health Syst. 2019 Jun 28;13:47. doi: 10.1186/s13033-019-0300-0 (PMC6598358; doi:10.1186/s13033-019-0300-0)
Supplement: Supplementary file 1 — Additional file 1. Theoretical domains framework and discussion guide. [file 13033_2019_300_MOESM1_ESM.pdf]

Current needs for the improved management of depressive disorder in community healthcare centres, Shenzhen, China: a view from primary care medical leaders

Kendall Searle, Grant Blashki, Ritsuko Kakuma, Hui Yang, Yuanlin Zhao, Harry Minas

Additional file 1
Theoretical Domains Framework and Discussion Guide

*Cane J, O'Connor D, Michie S. Validation of the theoretical domains framework for use in behavior change and implementation research. Implement Sci. 2012;7:37

TDF Domains and definitions*	Questions from Phase I Discussion Guide Relating to Each Domain
**Knowledge**<br>An awareness of the existence of something	Do you ever see patients like this? In what ways are they similar or different to those you generally see?<br>Can you give me a sense for how many you might see in an average working week? And what % of the total number of patients would that be?
**Optimism**<br>The confidence that things will happen for the best, or that desired goals will be attained	What % of these types of patients would you estimate go untreated?<br>Why is that the case?
**Beliefs about consequences**<br>Acceptance of the truth, reality, or validity about outcomes of behavior in a given situation	In your view, are patients like this considered a health priority in your clinic?
**Memory, attention and decision processes**<br>The ability to retain information, focus selectively on aspects of environment and choose between two or more alternatives	When treating a patient like this, what guidelines (if any) do you usually follow?
**Skills**<br>An ability or proficiency acquired through practice	How do you normally assess patients with depressive disorder?<br>How do you normally manage patients with depressive disorder?<br>To whom do you usually refer?<br>How do you normally follow-up patients with depressive disorder?

**Beliefs about capabilities**<br>Acceptance of the truth, reality, or validity about an ability, talent, or facility that a person can put to constructive use	Thinking about the three steps (assessment, management and follow-up) together, what problems have you encountered in patients with depressive disorder?
**Social/professional role and identity**<br>A coherent set of behaviors and displayed personal qualities of an individual in a social or work setting	Generally, speaking, what diagnoses would you consider for a patient like this? What else would you consider?
**Emotion**<br>A complex reaction pattern, involving experiential, behavioral and psychological elements, by which the individual attempts to deal with a personally significant matter or event	What factors, personal to you, do you think interfere with this intention (to treat depression patients)?
**Environmental context and resources**<br>Any circumstances of a person's situation or environment that discourages or encourages the development of skills and abilities, independence, social competence and adaptive behavior	What measures if any are available to your clinic to support patients like this?<br>In what ways, does your clinic help or hinder you, when it comes to diagnosing and treating patients like this?<br>In what ways, do your work colleagues help or hinder you in diagnosing and treating patients like this?
**Social Influences**<br>Those interpersonal processes that can cause an individual to change their thoughts, feelings or behaviors	In what ways, (if any) do the attitudes of society in general help or hinder you? And what about the attitudes of the local community?<br>In what ways, does the attitude of the patient help or hinder you? How is patient attitude influenced by their employer/family/friends?
**Behavioral regulation**<br>Anything aimed at managing or changing objectively observed or measured actions	What health policies (if any) are you aware of, help or hinder you with caring for these types of patients?
**Reinforcement**<br>Increasing the probability of a response by arranging a dependent relationship, or contingency, between response and given stimulus	What would help you to better assess, manage and follow-up this sort of patient?<br>What factors, personal to you, and your working situation, would encourage you to prioritize the care of these patients?<br>If you had to summarize your top three needs, to help you in your role as a primary care leader to improve the treatment of depression in your clinic, what would they be?
**Intentions**<br>A conscious decision to perform a behavior or a resolve to act in a certain way	Do you generally intend to detect, diagnose and treat depression inpatients attending your clinic?
**Goals**<br>Mental representation of outcomes or end states that an individual wants to achieve	With so many responsibilities, do you personally consider depression care to be a priority?
