# Supplementary material for: Current needs for the improved management of depressive disorder in community healthcare centres, Shenzhen, China: a view from primary care medical leaders
Source: Int J Ment Health Syst. 2019 Jun 28;13:47. doi: 10.1186/s13033-019-0300-0 (PMC6598358; doi:10.1186/s13033-019-0300-0)
Supplement: Supplementary file 2 — Additional file 2. Quotes in Mandarin Chinese and English translation. [file 13033_2019_300_MOESM2_ESM.pdf]

Current needs for the improved management of depressive disorder in community healthcare centres, Shenzhen, China: a view from primary care medical leaders

Kendall Searle, Grant Blashki, Ritsuko Kakuma, Hui Yang, Yuanlin Zhao, Harry Minas

Additional file 2
Quotes in Mandarin Chinese and English Translation

The following are original Mandarin Chinese and English translations of quotes included in the paper.

TDF Domain 1: Knowledge (Awareness of Depression)

Doctors depression aware but do not actively diagnose

应该绝大部分社康很少诊断抑郁症的。基本上我们社康就没有。假如我们怀疑他有这方面问题的时候，我们心里有数，但不会给他做抑郁症的诊断。

> Most community health service centers rarely diagnose depression, and our center doesn't diagnose it basically. We won't diagnose the patient as depression even though we suspect it. (D14)

Doctors rarely privy to patients' feelings

他只会说："哎呀，我心情很不好啊！我很压抑啊！我觉得生活没有意义啊！"但是里面的原因他不会讲太多。他只会跟信任的人讲，但是跟我们他可能觉得是一个外人，他不一定会讲得这么深刻。

> He will only say, "Ah! I'm in bad mood! I'm depressed! I feel life is meaningless!" But he won't talk too much about the internal reasons. He will only tell the ones who he trusts; but to us he may feel (the doctor) is an outsider, (and) he may not talk so deeply. (D01)

Patients regularly present with insomnia and somatic symptoms

讲到说疲劳、失眠、情绪波动，这种的病人会跟你说。比如说很多那种失眠的会过来，会看一下主诉失眠，有可能问一问就会问出这种情况，比如说焦虑。

> Patients may tell you that they have symptoms of fatigue, insomnia and mood fluctuation. For instance, many patients present with the chief complaint of insomnia, and perhaps we can consider it as anxiety after inquiry. (D16)

这样的病人来的时候，不会直接因为这个原因而来，反而是由于身体上的不舒服，例如失眠、劳累啊，来找我们。

Patients come to see us directly not because of mental reason. They come to see us due to discomfort such as insomnia, fatigue and so on. (D07)

TDF Domain 2: Optimism

Doctors perceive a sizable mental health treatment gap

我们后面的支撑团队，我们不像澳洲，澳洲有大量的心理医生、大量的团队在支撑着全科医生可以做。现在我们有资质的医生(name removed)，全科医生把这么多的病人筛出来我往哪儿放？因为我也知道有20%的比例都有抑郁症，焦虑症的比例也很高。但我能转到哪里去？深圳2000万人口就一个康宁医院，一个康宁医院就几十个医生。这2000万的20%是多少？要塞几百万的病人出来，医生怎么看的过来？

There are many psychologists in Australia and a strong team can support general practitioners to do their jobs. On the contrary, we have very few psychologists with certifications. If general practitioners screen all these depression patients out, what should we do? I know that 20% people have depression, and the proportion of anxiety disorder is also very high. The reality is, where should we refer these patients to? As you see, there are 20 million people in Shenzhen and there is only one Kangning Hospital and dozens of physicians inside. You can calculate the number, 20% of 20 million. How should physicians deal with so huge a number of depression patients? (D09)

TDF Domain 3: Belief about Consequences

Depression is not considered to be a treatment priority

优先应该没有，因为我们都是一致对待的。所有病人来……不能说你来，我就要给你特殊的照顾。没有。

There shouldn't be any priority, since we treat all (patients) consistently. All patients come…(It) can't be (that) you {the patient who has mental problem come (to the clinic), (and) I'll just need to give you special care. There is no (such thing). (D06)

嗯，脑袋里面还没有。因为我们是叫号系统的。一进来是拿号的，除非是那种急诊的，发高烧、外伤、肚子痛、有生命危险的，才会优先看。这种心理疾病还没有到那种程度。

Hmm, there is no (such concept) in (my) mind because we have a number calling system. (The patient needs to) take a number when they arrive, unless it's an emergency (case), (with) high fever, injury, stomach ache, (or) life-threatening (cases) will be checked first. These types of psychological diseases haven't reached that level. (D04)

TDF Domain 4: Memory Attention and Decision Processes

No standardized guidelines for the management of depression at clinic level

其实每年都会有一些指南，比如说加拿大的、美国的，但是到我们中国之后，就会经

过国内专家的解读，会制定一个国内版本，叫专家共识。这个国内版本会下发到每个医院，医院里也没有一个统一要求规定一定要按照专家共识的方法去治疗，只是专家认为要这样治疗，至于医生要不要根据专家的意见走就医生决定了。会的。有时候专家共识发布会在杂志上，通过杂志的流通，基层的医疗医生就会借助杂志里面的专家共识来提高自己的技能知识。

> In fact, new guidelines are issued in Canada and U.S. every year. However, experts in China read these guidelines and make a domestic edition, and we call it Expert Consensus. This edition is sent to every hospital, but there is no requirement that we have to treat patients according to this consensus. Experts believe that we should treat patients in this way, but whether to adopt it depends on each physician. Yes. Sometimes, this consensus is issued in journals, and physicians in primary hospitals can improve their techniques and knowledge by reading such journals (D14)

没有，都是根据医生的临床经验判断。所以说目前我们中国，社康在心理疾病诊断这一块普遍都不专业。有些中医医生对这一块还感兴趣，西医医生对于这一块的投入精力比较少、主观上也不是很重视。

> No. We assess according to physician’s clinical experience. Therefore, community health service centers are not professional in terms of mental disease diagnosis currently. Some doctors of TCM are interested in this aspect, but doctors of western medicine don't put so many efforts into it. Besides, they don't pay attention to it so much. (D13)

Doctors play a key role in general counselling and lifestyle advice

所以她就是人生有这么多不如意的地方，所以才导致她这个情绪这样的。我会告诉她就是……就是换一种想法啊，人人都有这样的情况，不是你单独就这样。我会告诉她这很普通的现象，然后就因为你的想法错了，所以才导致这个情绪这样低落。反正主要我会告诉她……，因为我毕竟学过一点点，所以我会告诉她如何去扭转这个局面，就是靠她自己的力量、自身力量看能不能换一种思维来扭转她的情绪低落。然后像抑郁啊，我们会告诉她，你要经常跟闺蜜说一说啊，找朋友啊，然后出门晒晒太阳啊，或者说去跑跑步啊、锻炼一下啦，这些方法来先纠正一下她那个。我说如果再不行的话，过一个月如果你感觉还是这个样的话，那我就会给你开一个、专门去找心理咨询师来谈。我会这样。

> So her emotions are consequences of so many unsatisfied situations in (her) life. I will tell her like…like to change a way of thinking, (such as) everyone has situations like this, (and) you are not alone. I will tell her this is a common thing, (and) then it’s just because your thinking is wrong that you feel depressed. I will mainly tell her regardless that…, since I’ve after all studied a bit of (psychological counselling), so I will tell her how to turn this around, depending on her own strengths, her own strengths to see if she can switch to another way of thinking to change around her depression. Then like depression, we’ll tell her (that) you need to talk to your best friends, to look for {hang out with} friends, then to get some sunshine, or to have a run and exercise a bit, and so on to correct her that (way of thinking). I say if nothing works, (and) you feel the same in one month, then I will write you a (referral) to specially seek for psychological counsellor for an appointment. I will do these (steps).(D03)

我们就会跟病人沟通，可能他存在忧郁症的问题，会告诉他忧郁症是什么，中国最担心的问题病人排斥，所以我们必须要花很多的时间跟他沟通，要让他认识到忧郁症是一个常见的病，就像感冒一样，是一个常见的、普通的病，也是身体哪一个地方遇到问题的正向。让他认识到一些疾病需要调整，需要生活方面的改善、个人调整或者是我们给他一个心理辅导就能解决。解决不了的就要吃一些药物，我们就要开始给他药物。

> We communicate with the patient firstly. If he/she has depression, we'll tell him/her what depression is. What we are afraid of the most in China is patient's rejection. Therefore, we must spend much time on the communication with patients and let them understand that depression is a common disease, which is just like cold. Besides, we need to let them know that some diseases can be cured via life style improvement, individual adjustment or psychological guidance. If it can't be solved by above-mentioned methods, we will prescribe some drugs for patients. (D08)

Doctors will use TCM either separately or in combination with Western medicine

首先就是要看病人愿不愿意吃中药，有的人来并不是来看中医的，是你发现了这个问题，你要问他愿不愿意吃中药。如果愿意吃中药，我会根据他的主要特征进行调理，会告诉他吃了这个药后看他的症状怎么样，有好转继续来、严重了建议他去上级医院去。中药在缓解这一方面有效果，但是不会有彻底的效果。如果他来的时候就是看中医的，我们肯定是会给他一些中医的治疗，会让他吃中药。如果是失眠的话，会给他贴耳穴、针灸的配合。其实主要是针对于失眠、头痛这一类的会有针刺疗法，我们会开中药然后配合这些疗法一起双管齐下。

> It depends on patients' willingness to accept traditional Chinese medicine. Some patients don't come to see traditional Chinese physicians, but we discover their problems, so we ask whether they are willing to take traditional Chinese medicine. If yes, we recuperate their body according to their symptoms. After that, I tell them to come to see me again if symptoms become better, and refer them to superior hospitals if symptoms get worse. Traditional Chinese medicine is effective in relieving symptoms, but can't cure it radically. If they come to see traditional Chinese physicians on purpose, definitely, we prescribe Chinese medicine to them. If they suffer insomnia, we use auricular application and acupuncture for them. In fact, there is acupuncture therapy for insomnia and headache, and we prescribe traditional Chinese medicine in combination with these therapies. (D16)

TDF Domain 5: Diagnostic Skills

Limited awareness and use of depression scales/screeners

可能我们国内的比较死板，如果你拿了一个表，直接按问题去问别人，可能不会妥当，别人会反感。我们在沟通的时候，其实基本上也在聊，但看诊的时候需要运用技巧把问题转成自己的话，不要一条一条的去问，大概的问了情况你就能评估病人.

Perhaps the application of scales in China is somewhat rigid. It's not proper to take the scale to ask patients questions directly because they may feel disgusted. Basically, we talk with patients with some skills in the communication, not just ask questions rigidly. You can assess patients after knowing the general situation. (D02)

Doctors pessimistic about screener utility and effectiveness

现在我们用的还是中国通用的简易量表，因为现在量表很多，用起来也不是特别的方便，有一些量表的问题很多，需要花费的时间非常长，孩子来看病往往是课间来的，再多十分钟他就上课了。所以他不能完全把它填完，他一看有那么多页就不做了，所以需要一个简单点的。比如说只包含主要的项目，他能够在迅速的把它做完，这个可能是18-30题就适合他，5、6分钟左右的时间，超过十分钟他会觉得好长，心里面也着急，心想都快上课了，下次再做吧，但下次他可能就不会再回来了。

Now we use simple scale commonly used in China. Now there are many scales and it's not very convenient to use them. Some scales have a lot of questions and much time is in need to finish them. Students use their time between lessons to see us and they'll be late when finishing the scale. They refuse to fill the scale when they find it very long. That's why we need a simple one with main questions, 18-30 questions. It takes them 5-6 minutes, while10-minute a scale is too long for them, as they want to go back to class. If ask them to fill the long scale the next time, they may not come back forever. (D14)

但很多时候我们觉得这个量表并不能反映真实的情况，因为我们的这些病人太聪明了，他们看到量表就知道这个是干什么的，他会根据他自己的愿望来填写这个量表，比如说他希望医生觉得他严重，他就会把选项向严重的情况勾选。如果他认为会造成不好的影响，他就会勾选程度轻的选项，他会通过他的主观判断来选择性的回答量表的问题，所以不见得能够真实的反映他的问题，医生在后期的心理咨询当中会发现这个病人的情况远不是他填写的那么严重或者是比他填写还要严重，但他有他的苦衷、他有他的想法，有时候他会有一些顾虑，我们也表示理解。

However, we feel that the scale can't represent the reality, as our patients are very smart and they know what the scale is for. They fill the scale according to their wishes. For instance, when they wish physicians to feel the severity of their disease, they tick the serious options; when they want to avoid negative outcomes, they select mild options. In a word, they tick answers according to their subjective wishes, rather than their real status. In later psychological consulting, physicians find that their status is not so severe as or more severe than the scale outcome. They have their difficulties, ideas and concerns, and we understand. (D14)

Doctors Actively Choose Time Appropriate Tools To Support Diagnosis

所以经常用PHQ-2，如果PHQ-2有3分以上说明有问题，就会用PHQ-9再筛一遍。如果PHQ-9达到诊断的标准就会转诊。如果很轻度的，就让他多来几次，约在病人不多的时候过来。因为看病时间控制的很紧，只能约病人少而且他有空的时候。

…that's why we use PHQ-2 very often. There are problems if the score of PHQ-2 is higher than 3, so we use PHQ-9 to make another evaluation. If the result of

PHQ-9 evaluation also meets the criteria, we refer patients to other hospitals. As for mild patients, we ask them to visit us for several times when there are not many patients, as we have to control the counselling time for every patient when we are busy. (D10)

他是美国的一个家庭医生。他教过我们那个方法，我觉得挺好记，以前我们只是客观的让病人去自评多少分，现在就不是的。比较轻的我们就不评分了，他就教我们SIGECAPS，C是注意力、I是兴趣，还有G是内疚等等，判断的方法都在这上面，我们就自己掌握住了，这就是算唯一国外的东西。

There is a family doctor from the United States. He taught us the method and I feel that it's easy to remember. Previously, we only made patients give score to themselves, but now it's different. We don't assess patients with mild depression by using the scale. What the family doctor teaches us is SIGECAPS, in which C means concentration, I is interest and G is guilty. The assessment methods are what I mentioned above and we have mastered them. It is the only international reference we use. (D08)

TDF Domain 6: Beliefs about Capabilities

Drs receive limited professional development

治疗方面就是药物的问题，我们使用药物很受限制。在中国很多药是不能开的，第二个就是治疗手段方面，这一方面的培训不多，医生没有很大的把握去给他做治疗。不知道做给他治疗是对的，还是说不对的，怕误导或者说耽误他。

In terms of therapies, there are issues of drugs. Drug use in our center is limited. Many drugs can't be prescribed in China. Besides, with respect to treatment means, training about it is rare. Physicians are not so confident in treating patients like this, and don't know what therapies are proper for the patients. We are afraid of delaying patient's illness. (D11)

Drs' confidence in their ability to treat is low

遇到过困难？就是……，嗯，第一个困难就是自己的。就是自己觉得很不擅长，很不擅长来、来治疗。哎呦！遇到这个好像不知道怎么、怎么弄好！应该要跟他说一些什么话？这些都是需要学习一些专、比较专科的问题啊！第二个可能，第一个困难就是医生自己方面啦。

Encountered any problems? It's like…, hmm, the first difficulty is my own. It's like I don't think I'm good at (treating mental problems), not good at the treatment at all. Ah! It's like (I) don't know how, how to do it well when come across this (problem)! What should (I) say to him {the patient},? These all require studies of some specialized questions! (D05)

TDF Domain 7: Social/Professional Role and Identity

Doctors are not psychiatrists

嗯……药物！抗抑郁药、抗焦虑药，我们那儿没有。我们医院也许有，但是因为呢……，我不想、我不想轻易的下这个诊断，不想轻易的开这个药。如果病人在康宁他下了这个诊断，然后需要长期服用这个药，那我可以为他调拨这个药过来。但是我不想说我先下诊断，因为毕竟我

不是精神科医生。虽然有心理咨询师证，但是我们国家是要……二级、我们是要二级心理咨询师才能够开这个药。

> Hmm…medicine! We don’t have anti-depression and anti-anxiety medicine. There might be (such medicine) at our hospital, but because…, I don’t want to, I don’t want to easily make this diagnosis, to easily prescribe this medicine. If the patient is made this diagnosis (as depression) at Kangning Hospital, (and) then (the patient) needs to take this medicine in the long run, then I can transfer this medicine over. But I don’t want to first make this diagnosis, because after all I’m not a psychiatrist. Although I have the psychological counsellor certificate, but in our country it requires…Class Two, we need to be the Class Two Psychological Counsellor in order to prescribe this medicine.(D03)

Doctors actively avoid stigmatizing patients with a depression diagnosis

很多时候病人很抑郁过来看诊，你不会告诉他是抑郁症的，他很抗拒的。因为中国人的文化很抗拒这种精神、心理疾病。很多时候跟他聊一聊是什么原因造成，可能是什么情况，侧面地告诉他得的是抑郁症。但是不同的人群可能不一样，这一片区的人群接受程度会稍微好一点。你可能到别的社区，可能就不太接受，总觉得给他扣上抑郁症这个帽子或者是心理疾病的帽子，他很不喜欢。

> In many cases, you don't tell the patient that he has depression when he comes to see a doctor. He is (usually) very resistant, since the Chinese culture is very resistant to this type of mental, psychological disease. In many situations (the doctor) talks to him [the patient] about what the causes are, what might the situation be, and tell him [the patient] indirectly that (he) has depression. But different groups of people might (react) differently. The people in this community might accept it slightly better, (but people from) other communities might not really accept it. He [one from other communities] feels he is regardless put on a hat of depression or psychological disease, and (might) dislike it very much.

不过我们还是会比较慎重，因为有些人对于这个比较忌讳，他觉得面子上不好看。我们在怀疑这个病的时候也不会轻易的说出来。就算我们很怀疑的，也可能婉转的跟他说一下。我自己会说，你最近压力比较大，一不注意就会得抑郁的。别的医生可能会说“你再不注意可能就有抑郁症了”。但很多时候我们一说，你压力不能太大，要调节一下，有些病人就会主动问，我有抑郁症吗？一般这样的人就比较轻一些。也有个别的，你要这样说的话，他会不高兴。那样的病人我们建议家属带他去专科医院，也不敢说让他去专科医院，精神病医院太严重了，让他去综合医院，大的综合医院有心理科的。

> We are cautious for the diagnosis because it’s a taboo for some people, and they may feel embarrassed. We also don’t say it out easily when suspecting that it's depression. Perhaps we’ll tell the patient euphemistically. Maybe I’ll tell patients that their pressure recently is somewhat great, and it will be possible for them to get depression if they don’t pay attention to it. Some other physicians may say “It’s possible for you to get depression if you don’t pay attention to it.” But most of the time, we’ll tell patients that their pressure should not be too great and they

need to adjust it. Some patients may initiatively ask us whether they have depression or not, and severity of these patients is not so high typically. But some individual patients will feel unhappy if you say above-mentioned content to them. For these patients, we advise their family to take them to go to other hospitals. We dare not to recommend specialized hospital dedicated to mental diseases, but recommend general hospitals because there is psychological department in large general hospitals. (D13)

大部分的时候我们不会诊断他抑郁症，一方面他可能对抑郁症比较恐惧，因为我们在电视报道上或者是新闻报道上，都说这个病很难治疗，会带来不好的后果。所以大部分时候我们会给他诊断神经衰弱，可能跟大家交流起来相对困难一点，这样他的老师不会觉得他患病特别严重，他自己也不会觉得自己得了什么严重的疾病。

In most cases, we don't diagnose depression for them. The main reason is that they are very afraid of depression, as they feel this disease intractable and it will bring negative outcomes, based on information from TV and other types of media. Therefore, we diagnose their symptoms as neurasthenia; and, it would be a little difficult to communicate with them. In this way, neither their teachers nor themselves feel their disease severe. (D14)

TDF Domain 8: Emotion

Doctors fear making mistakes or being confronted with conflict

这些是要、为什么要、要、要设立一个精神病的管理那一块，那个公共卫生，就是因为它那种也是属于病程比较长的疾病嘛！不是说你几天就能解决的，需要长期的管理。管理得好的话可能就好一点，管理得不好也很多也有一些。也很怕那些肇事的啊！就是那些精神病患者，他也、有时候会发作，或者没按时吃药，或者什么的，他就、有一些什么刺激一下他，他可能就发作了、就肇事。就可能很麻烦！所以，很多……很多医生管的这些其实也心惊胆战的，因为（笑），因为自己……，怎么说呢也不是这个专科的，又不太那个，又不太熟悉这个领域，所以管起来还是比较困难，觉得压力挺大。

These need to, why (it) needs, needs, needs to set up an area for mental health management, (for) that public health, it's just because it belongs to a kind of disease that has quite long courses! It's not like it will be solved in a few days, but rather it needs long term management. It {the patient's illness} might get a bit better under good management, (and) there are many (cases) or some (cases) that (the illness) is not well managed. (The doctors) are also very afraid of those who cause accidents! Like those mental health patients, he {the patient} also, sometimes will fall ill, or doesn't take medicine in time, or something like that, (and) he will just, might just fall ill, and cause accidents if there is any stimulation. It will be very troublesome! So, many…many doctors are frightened to manage these (patients), since [laughing], since (the doctors) themselves…, how to put it, like (the doctors) are not specialized in this, and are not very that, not very familiar with this area, so it's rather difficult when (they) manage (the patients), (and) feel the pressure is big. (D05)

Doctors are not attuned to providing psychotherapy

嗯……我除了觉得时间，还有……呃，还有一点个人因素，就是不愿意往这方面去发展。因为我们在培训的时候，老师经常说，心理咨询师也要有自己的导师，也要有自己能吐槽的地方。但是我目前没有这种导师，所以我就不想去接受这么多……。因为他们必然披露的事实有时候是虐待啊，有时候是家暴啊，有时候什么之类的。我觉得我接触到太多这样的负暗、就是负性的这些阴暗面，我觉得我、我的情感也会受到牵连。所以我就不想自己被他们给带入到这种阴暗面上，因为我没有我的吐槽老师吗。所以我就不想继续往这些方面发展下去。这就是我为什么也不愿意给他们提供、进一步治疗的原因。

> Hmm…I think in addition to time, there is also…eh, there is also a bit of personal factor which is (I) am not willing to develop in this direction. Since when we were under training, the teacher often said, psychological counsellors also needed to have (their) own supervisors (and) need to have somewhere that {someone who} (they) could make complaints (to). But I currently don't have a supervisor like this, so I don't want to receive so much… Because the truth they reveal must be sometimes torture, (and) sometimes domestic violence, and sometimes some (other) types of (unpleasant things)… I think (if) I come across too much this kind of negative darkness, like these negative darkness sides, I think I, my emotions will also be affected. So I don't want to be taken into this type of darkness by them {the patients}, since I don't have my supervisor to complain to. So I don't want to continue down on these directions. This is why I'm also not willing to provide them further treatment.(D03)

TDF Domain 9: Environmental (Health System) Context and Resource

High volume of patients and short consultation times

第一个对于发现病人抑郁这一块是有一定的欠缺，有可能也会遗漏，比方说经常头痛、失眠的病人，并不是他的头有什么器质性的问题，他有可能是焦虑、抑郁。我们平时看病的时候病人太多了，可能五分钟要处理一个病人，有可能不到五分钟，2、3分钟就要看一个病人。根本没有时间问他的病史，也不可能有这个意识关注他的心理方面的问题，所以这个有可能是遗漏的地方。

> Firstly, there is deficiency in the aspect of depression finding, and perhaps patients with depression might be omitted. For instance, some patients who usually have headache and insomnia may have no organic issue, and they may have anxiety and depression. Commonly, we have too many patients, and perhaps we need to treat a patient every 2-3 minutes. We have no time to ask his medical history at all, and it's also impossible to concern whether the patient has psychological problems or not. This may be the omitted aspect. (D12)

Limited trained mental health resource at CHC level

其实看医生的。在社区里面，全科医生的技能中应对抑郁症的只占很少，包括其他的心理疾病如焦虑等，医生能做的事情不多。很多医生都不能完全搞清抑郁症的诊断，什么时候需要筛查也不知道，大多数的医生都是这样。如果有一个简单有效的量表，能花个1分钟以内或者是30秒左右就能筛查，对于医生的帮助很大。

> Actually, they come to see physicians. However, only a few physicians in community hospitals are able to deal with depression. They can't do much for

psychological diseases like anxiety. Many or most physicians don’t know how to diagnose depression or when to screen. It’s very helpful if there is a simple but effective scale to screen patients in a minute or 30 seconds. (D10)

Limited trained mental health resource at Hospital level

有时候病人需要四点钟要去排队，病人又很多，医生能给病人看病的时间又很短，早期还没有湘雅二院，那时候我给转诊的病人，他们基本都不再去的。因为排队很麻烦，有些好不容易看完病排完队了，开完药回来发现又有副作用。所以基本上我转诊到康宁医院的都是拒绝的，最后都没有得到很好的结果。

> Sometimes patients need to queue up from 4 o’clock. Besides, physician’s time for each patient is quite short because there are so many patients. When the second Xiangya Hospital of Central South University was not our guider, patients that I referred to Kangning Hospital didn’t go to the hospital basically because queuing is too troublesome. Some patients might be diagnosed after queuing with great efforts, and then found that the prescribed drugs had side effects. Therefore, most patients that I referred to Kangning Hospital refused to receive further treatment in the hospital, and no good results were received finally (D08).

嗯，然后医生得到的知识、本来因为我们中国的心理健康还没纳入到全科医学之中、这个里面，还是算是专科的。而专科的我们一面、心理医生、精神科医生都非常少。我自己就曾经去过精神、精神、就是康宁医院，我去了解过。嗯，医生就那么几个，病人几层楼。啊！就是大群大群的，一个人管大群大群的人。所以你照、顾及不来。其实病人是非常需要有这些人帮助他。你像那种协疗、社会工作者、心理顾问啊，这些社会上有！像真正的话有这些医疗机构、这些不是医疗机构，就是协疗、协同协作者。但是他们往往收费很贵。所以我们中国的老百姓，普通的还一般接受、没办法去接受。

> Hmm, then the knowledge that doctors receive {have learned} (does not involve mental health), to start with because in our China, mental health has not been included in GP studies, (and) it still counts as (a) specialist (area). However, in terms of (mental health studies being a) specialist (area of study), on one side, the psychological doctors, (or) psychiatrists are all very few. I have personally been to the psychia-, psychia-, like Kangning Hospital, to learn about (them). Hmm, there are only a few doctors, (but) the patients are packed over several floors. Ah! It’s like large groups (of patients), (and) one person {doctor} looks after large groups of people {patients}. So you cannot look, manage (all the patients). In fact the patient badly need help from these people {doctors}. There are that kind of assisted treatment, social workers, (and) psychological counselors in the society! But the real (organization that provides specialized treatment) like these medical institutions, these (the above mentioned assisted treatment, social workers, and psychological counselors) are not medical institutions, (they are) just like assisted treatment, the collaborators who work together (along with the medical institutions). (D04)

Patients lost to a developing referral system

一般我感觉病人比较严重的，会转到医院那边去，转过去后我们很少再跟进了。一般来说后续的跟进，都是医院那边已经确诊了抑郁，医院会把病人转回来社康，然后会告诉我你负责的这个小区有这样的一位病人，我们才会跟踪、随访。如果他的程度没有达到医院诊断的条件，我们这边可能没有再过多的随访。

When we feel that their status is somewhat severe, we refer them to hospitals, and we seldom trace them after the referral. If the referred hospital has confirmed the diagnosis of depression, patient information would be sent back to community healthcare center and they tell us that there is such a patient in our community. Only under this circumstance would we trace them and follow them up. If their severity hasn't met the diagnosis criteria, we don't follow them up. (D17)

Poor CHC ability to follow-up patients

没有再后续跟踪。如果再来的话会跟他聊一聊，没有的话就不聊了。因为这些人留的电话往往不准确，随访的时候发现打回去不是他本人。>> 比较困难。就看他自己的意愿，如果他真的要继续看，会到社康来。留的电话都是错误的，都是找不到他的。

We don't follow up patients further. We'll talk with them if they come again. Telephone numbers left by them are always incorrect, and the phone is always not answered by the patient when we conduct telephone follow-up. It's somewhat difficult. It mainly depends on the willingness of patients. They will come to our center again if they really want to treat it continuously. However, many telephone numbers left by them are incorrect, and thus we can't find them. (D07)

No antidepressants at clinic level

对，因为目前抗忧郁的常见药物当成精神病药物管理，很多领导认为你社区不能有的，但我们社区是最需要这样的药物。像以前的百忧解、赛乐特我们社康里面还能见到，现在一搞什么药都见不到了，很早我们用百忧解、赛乐特这个药物来抗忧郁，只有这两种药物。我们也没有别的选择，只有这两种药物，我们也是治疗有几个病人的，不是很多，但是效果都是挺好的。现在没有药物，我们要转诊到医院去，我们给他诊断出来了，我们还要转诊到医院去让医院给患者开药。

Currently, common drugs for anti-depression are managed as antipsychotics. Many leaders think community health service center should not have this kind of drugs. However, what our center requires the most is these drugs. Previously, there were Prozac and Seroxat in our center, but now there is nothing. We had no other choice but these two drugs for treating patients with depression. Though not so many patients received the treatment in our center, efficacy of these two drugs was quite good. Now our center has no drugs, and we need to refer the patients to hospitals. We can diagnose them and then refer them to hospitals for prescription. (D08)

因为我们在社康没有诊断能力、没有药品发放，有些人根本就没有见过我们这里的医生，所以跟你没有什么面对面的交际。他直接去康宁医院取的药，又恰好住在我这个片区，所以他的信息就到了我这里，我跟这个人根本就不认识，没有见过面，只是电话联络，所以有些人不愿意接受你的管理，因为他觉得不需要你的管理。

Community healthcare center has no diagnosis capability and no corresponding drugs for them. Some patients even haven't met physicians here, so there is no face-to-face interaction. They get drugs in Kangning Hospital and but coincidently live in this community, so their information is here. We don't know them and haven't seen them, and we just keep in touch by phone. Therefore, some of them are unwilling to accept our management, as they feel it unnecessary. (D16)

No designated consultation space for mental conditions

嗯……诊所，我们的诊所虽然关着门，但是不隔音。所以病人的隐私保护不了。你在里面跟病人说话，外面的病人听的一清二楚。所以出来以后人家一看，哦！是这个人啊！他心里有问题啊！那所以还是保护不了病人的隐私。这是一个障碍！

> Hmm…clinic, although the door is closed at our clinic, but it's not soundproofing. So the patient's privacy can't be protected. The conversation you have with the patient inside is heard clearly by the patients outside. So when (the patient inside) comes out, everyone will have a look, oh! It's this person! He has a psychological problem! Then so the patient's privacy can't be protected. This is an obstacle! (D03)

原来的地方环境特别糟糕，只有两个房间，人山人海，你里面还没有看完，人家已经堵在那里等着了。所以说原来的工作场景是不能支持的。有的甚至是连个拉帘都没有，有一些女性私密的部位要查看，还得找一个地方关起门来，所以比较困难。

> The environment of the centre, where I worked previously, was extremely terrible. There were only two rooms, (always) full of patients. Many patients waited for you in the room. Therefore, my previous working environment didn't help me with diagnosis. There wasn't even a curtain. When we needed to check a female's private parts, we had to find a place and close the door, which is somewhat difficult. (D07)

优先照顾的话，因为要注意保护隐私，例如举办活动我们每周要单独为他们挤出一个房间，然后和他们约一个时间与他们聊天。有时候我们会把家属约过来，大家聊一聊，家属到底是哪一块解决不了的，我们会找社工、找政府机构为他们提供帮助。比如说家里真的很穷，起码柴米油盐我们可以帮到他们，又例如说到医院挂不到号的，我们这边也可以提供帮助。所以我能帮病人做的，只能说单独一个下午约2、3家人或者是3、4家分开时间，每一家大概半小时去沟通。但是这种情况不多，因为我看的门诊病人已经够多了，社区还要单独留一个房间，所以现在我们会在最后的四分之一里面找比较难搞的那种病人做这个工作。

> For patients with depression, we need to pay attention to their privacy protection. For instance, we need to arrange a room for them for holding activities every week, and make an appointment with them for chatting. Sometimes we also ask their family to come to the center for solving their difficulties by asking the help of social workers and the government agencies. For example, if economic condition of the patient is really poor, we can help them in terms of daily necessities at least. If the patient can't register in the hospital, we can also provide assistance for them. Therefore, we can only arrange about half an hour for each patient for communication if there are 3-4 appointments. However, the situation is rare because we have so many patients to see, and we need to arrange a room for them separately, and thus we only select one intractable patient from them. (D17)

TDF Domain 10: Social Influences

Poor general/community health literacy

中国社会普通老百姓对于心理问题，大家觉得就是精神病、神经病，所以他不接受，导致老百姓不愿意去看病，这是由于社会大众对于这个病的认识不够，他不愿意来就诊，所以也导致了发现少的、另外一个原因，中国不像澳洲，他们有很多网站、报纸、免费电话可以去咨询。但在中国没有，他就闷着，越闷越厉害，病情就会加重了。

> In Chinese society, common people consider psychological problems as psychiatric problems or mental illnesses. They don't accept it, so they are unwilling to see physicians due to these issues. Common people don't have right knowledge to depression and are unwilling to consult a physician that's why we haven't found many patients with depression. Moreover, different from Australia, we don't have so many websites, newspaper, or free calls for consultation, so they don't talk with others about their problem, which worsens the illness. (D09)

Chinese underlying culture:- Loss of face accentuates poor health seeking

第一个，文化。我们讲中国文化和澳洲是不同的。这个中国的话，她觉得这算、这不算什么，然后这是自己的隐私。我说出来、什么自己是失败的母亲、不合格的妻子、不称职的员工，这是说、这是自己的过错。哦，我、就是认同。怕污名吗！不会说什么。中国人就是家丑不外扬。一般自己闷着了。啊，我们还有家庭暴力的，都会闷着的。并且还有老公不打自己就是觉得不重视自己，没受到关注的。这些都……。这种大环境。它的文化是这样。并且哩，我们中国毕竟还是在发展中国家。大多数为了生存，要活着，还没达到这种精神和心理的满足。啊！她觉得我这么闷着，我能干活，我还能挣钱。

> First, the culture. We say Chinese culture is different than Australia culture. According to Chinese culture, she thinks this is considered, considered as nothing, and this is her own privacy. If I {the patient} say something like I am a failed mother, an unqualified wife, an incompetent employee, this means, this is (the patient's) own mistake. Oh, I {the patient} then it means the patient just admits it's her own fault. Chinese people are afraid of stigma! (The Chinese patient) won't say anything. Chinese people are like let the household disgrace be buried inside the house. Usually (the Chinese people) keep it to themselves. Ah, we also have (patients who experience) domestic violence, (and they) all keep it to themselves. Moreover, there are even (wives who) think (they) are not valued or paid attention if (their) husbands don't beat them. These are all… This (is the) big environment. Its culture is like this. Moreover, our China after all is still a developing country. The majority (of people) are living for surviving, (and) they have not yet achieved the psychological and mental satisfaction. Ah! She feels I'll just keep it to myself like this, (and) I can still work, I can still make money.D04

我们中国是往往是被动式，比较……严重了。这个她还算是轻微的。不会来。她自我、自我调节。

> Our Chinese (patients) often are passive, (and wait until their illness is) rather…serious. This (patient condition) still counts as mild. (The Chinese patients) won't come. She (the Chinese patient) self, self regulate (her condition). (D04)

他们整天都很忙，他们在工厂的比较多。所以这种求助行为、找医生，他们可能大部分人会觉得能够自我调节，而且不认为是有可能是一个疾病。所以他们的求助行为，这个方面可能没有关内那么强烈。

They are very busy everyday, (and) many of them (work) at the factory. So this kind of help seeking behaviours, looking for the doctor, perhaps the majority of them think they are able to self-regulate, and don't' think this might be disease. So their help seeking behaviours, in this area might not be as strong as people from within the border. (D06)

Intense stigma associated with the main specialty hospital (Kangning)

像刚才说的康宁医院，有些人确实是有这一方面的问题，但觉得去到那里治疗就会有不好的名声，就说精神有问题才需要去到哪里。所以人家也不怎么愿意去。如果是阻碍的话，这一点应该是最主要的。另外家里、身边人的一些阻碍行为，这也是原因之一。

I mentioned Kangning Hospital just now. Some patients have depression exactly, but they are afraid of bad reputation if they go to Kangning Hospital. After all, it's widely regarded that only patients with psychological problem go to the hospital. Therefore, they are not so willing to go to the hospital. I think this is the major obstruction. Besides, interference from family and people around is also the obstruction. (D07)

而且大部分都可能没有接受恰当的治疗。第一，他不会来看医生，他不想就医治疗这方面的问题,怕告诉医生。第二，就算他来了告诉你了，有些人转诊出去后，他不一定去。在中国的文化中看精神科、看心理科都很忌讳，觉得是神经病，不好。

In most cases, patients don't receive proper treatments. There are several reasons. Firstly, they don't come to see physicians for depression, as they are unwilling to and afraid to tell physicians their problems. Secondly, when we want to refer them to other hospitals, they don't go there, as in Chinese culture, it's a taboo to see psychiatrists or psychologists, as most people are afraid of being considered as a psycho. (D10)

他们很多时候去看的时候，他就给了一个假的信息。特别是在康宁医院里面，他们通知我们说这是你社区里面的一些病人的时候，他们很多都是给的假的地址或者是假的联系方式，比如说他可能提供的是一个他朋友或者是其他人的信息时候，你很难再追踪到，既使是你知道那个是，如果是错的完全就没有了。

When patients go to see a doctor, they usually give false information, especially in Kangning Hospital. When doctors in the hospital tell us these patients of our community, but the address or contact information they give is false. They may provide the information is one of their friends or other people, and thus you are hard to trace. (D02)

Community induced isolation and discrimination

如果是让他们知道都是阻碍，所以尽量不让他们知道，因为他们会指手划脚的，我们就怕这种情况，也有可能会产生这种情况。尤其是喜欢八卦的人还是比较多，大家茶余饭后，你传我一下、我传他一下，最后传一传就病危了，尽量不让他们知道，我们很担心他们知道。如果让他们知道的话会是一个很大的阻碍作用。

It must be an obstacle if local community residents know it because they will make indiscreet remarks or criticisms. That's what we are afraid the most. Many people like to gossip with each other and the disease of the patient may be gossiped as critical illness finally. Therefore, we do our best to not let community residents know because it will be a great obstacle. (D08)

这个肯定是会有的。比方说我们去随访他们、去他们家里，附近的居民都会探出头来看一下，“怎么回事，怎么这么多人都来了，连警察都来了”。他们会觉得这个人是不是犯了什么罪、对于他另眼相看，多多少少增加了病人的心理压力。

> Absolutely. For instance, when we go to the patient’s home for follow-up, nearby residents may pop head out to see what happened and gossip because there are many people including policeman. They think the people we visit has committed a crime, which has increased the mental stress of the patient. (D12)

你家里人都不管你的话，那其他的人的话，我、我觉得中国人的话，远远没有像外国那么热情，就是可能、真正会有一些、会带有一些歧视的，这个是精神病哦！那个是……什么、心理有问题。可能还会有些人还会远离他。

> If even your family doesn’t care about you, then others, I, I think Chinese people are far less warm-hearted than foreigners, (and) just maybe, (the Chinese people) will truly have some, have some discrimination (over the patient), (and say) this (person) is a psycho! (And) that (person) is…something like psychologically faulty. Perhaps some people will even stay away from him {the patient}. (D05)

Family and friends can be important facilitators for patient care

家里人还是非常重要的，包括朋友，对于疾病的支持非常重要，包括他的服药。有家里人支持跟没有家里人支持的差距是真的非常远，当然很多时候这个病因为是长期的，有很多家人、朋友一开始非常支持，到后面可能就会太痛苦.

> Both family and friends are extremely important for supporting patients. It really has great difference between patients with support of family and those without the support. However, as duration of this disease is quite long, many family and friends may just support them at the beginning, and also feel torturous in the later stage. (D02)

如果是家庭……条件好，或者是健康意识好的话，他们主动去寻求帮助，就会替……哎，医生我儿子怎么怎么了，出现这种情况。你看有什么办法？总是亲人、一般都是亲人主动找医生。然后……那个，我一般都说，带你儿子过来看看。这种抑郁症的人是被推着来找医生的！

> If the family…condition is good, or (their) health awareness is good, they (the family) will actively seek for help, for…ah, doctor my son has such and such problem, (and) is having this kind of situation. Do you have any idea? It’s always family, usually it’s family who takes the initiative to look for the doctor. Then…that, I usually will say, bring your son over (and let me) have a look. This type of depressed people are pushed to the doctor (by their family)! (D04)

就是比如说，他、因为有些病人呢，他……一直在家里，比如说我不开心，或者不吃不喝。他有可能就是不会觉得自己得了什么疾病，但是可能在家属看来，因为他家人整天能够观察到，旁观者有可能会看得更清楚一点。如果家属能够意识到这个问题，而且还能带他来就诊，可能会、治疗起来会更理想一些。

> Just for example, he, since some patients, he {the patient}…stays at home all the time, for example I {the patient} am not happy, or don’t eat and drink. He possibly

doesn’t think he’s had any illness, but perhaps from the family’s perspective, since the family can observe (the patient’s behaviours) everyday, (so) the bystander might see it clearer. If the family can realize this problem, and can bring him {the patient} over to see the doctor, (then) it might be, be more ideal for the treatment. (D06)

Poor family understanding of depression can lead to poor treatment outcomes

我就是刚才说了人们歧视。人们歧视不光是大众的歧视，那可能还有老板或者他同事对他的歧视。还有家人也不理解，他觉得你怎么会、怎么会有这种问题？**…**。有的人家人他根本没有这样的常识。他没有这样的常识，你让他怎么样去理解他？他肯定很难去理解他。家人就会说，你不要给我打电话，我家里没有这样的……精神、精神有问题的。他也觉得一个家人如果有了这样的一些精神有问题的，他就是一个非常羞耻的事情。

> I just mentioned that people discriminate (the patients). People’s discrimination does not only mean the discrimination from the public, it could also include the discrimination from the boss or his {the patient’s} colleagues. Even family (sometimes) doesn’t understand, (and) he {the family member} thinks why do you, why do you have this type of problem?….Some family member he doesn’t have this sort of common knowledge at all. (Since) he doesn't have this sort of common knowledge, how could you ask him to understand him {the patient}? He {the family member} will surely find it hard to understand him {the patient}.The family will say, you don’t call me, (because) I don’t have this (family member)… with mental, mental problems at home. He also feels if he has a family member who has such mental problems, this will be a very shameful thing. (D01)

会，肯定是跟他的家庭、跟周围的环境有关系。如果家里都是很积极的就不怕，你有焦虑、有抑郁你去看病，你去看病、去看医生吃完药就好了，如果有些人觉得家丑不能外扬，你这个情况神经兮兮的不能去看，就更糟糕了。

> Yes, their family members and environment around definitely have impact on them. If family members are all very positive, there is no problem. They encourage patients to see physicians because of anxiety or depression, as they feel that problems can be solved after taking medicine. If family members consider it as a scandal and keep it in secret, the status would worsen. (D09)

Poor employer attitudes towards depression

首先一个，就诊的时间。因为、因为你频繁的请、你请假首先要通过雇主的同意。他们雇主的话会有一系列的请假制度，比如说你……少上一个班要扣多少钱啊！或者说我最近时间、我工厂在赶期，我不准你请假。啊，对！有可能会存在这个问题。就是他有可能会影响到他的就诊时间。

> First of all, the time to see the doctor. Because, because you {the patient} frequently ask, you need to obtain the employer’s agreement first (if) you (would like to) ask for leave. The employers have a system for leave, for example you…how much money will be deducted for working one shift less! Or I {the employer} recently, my factory is catching up (to the deadline), (and) I do not allow you to leave. Ah, yes! Possibly there will be this problem. Just like he {the employer} possibly will affect his {the patient’s} treatment time. (D06)

不一定是雇主，这个社会总体的态度是不好的，都不会主动的说明心理问题。比如说我也不会说，觉得今天好像心理有点问题了，然后去跟老板说能不能请个假看一看医生，他说什么病，

如果你说发烧了，老板可能会痛快就批假了。但如果你说你觉得有点睡不着、情绪不是很好想看医生，老板会说“乱想什么，好好的干活”。

> Not only the employer, the overall attitude of society is not so good. No one will just open-up and tell others that they have some psychological problems, including me. For instance, when you ask your employee for leave, he/she may ask you your problem, the employee may approve it rapidly if you say you have a fever or something else. However, if you tell him/her that you have a poor sleep or bad mood, he/she may advise you not to think too much and work harder. (D02)

如果他私底下周末去看康宁医院或者是什么样的，老板不看你的病历，他根本不知道的啊！你正常人都有休息的时候吗！我觉得他们应该是在工作当中，他们因为焦虑症这种，工作当中应该表现得更积极，做什么事都立刻就做完或者怎么样的，没影响到他的工作。所以周围人也许只是觉得他性格毛躁了一点，也不存在说那种。

> If he goes to Kangning Hospital over the weekend in private or something like that, the boss wouldn’t know at all if he doesn’t look at your medical records! You normal people even have time for rest! I think they {the patients} because of the anxiety, should perform even better at work, at work, (such as) complete tasks very quickly when they are at something or something like that, (and) it {the anxiety} hasn’t affected his work. So people around him might just feel he is a bit anxious, and there doesn’t exist that kind of (suspicion that the patient has mental problems). (D03)

嗯……应该会有影响。因为呢，有一些，比如说有一些单位哦，特别一些私人的，啊，你有这一方面的问题，啊，你让他老板知道，他可能就想方设法把他炒了或者是什么。他们病人其实也、也害怕，也害怕让人、为什么？怕让人知道。就是第一个，怕人家歧视他。第二，就是怕会不会跟他的一些生活啊、工作啊、还有学习，会不会受到影响。嗯！如果是工友的话，自己的事情也不太愿意跟别人讲，因为也怕它传播到整个工厂里去嘛！

> Hmm…there should be such influence. Because, some, for example at some workplaces, especially some private ones, ah, (when) you have problems in this area, ah, (and) you {the patient} allow the boss to find out about it, he {the boss} might take whatever he can to fire him {the patient} or something. The patients in fact also, also are afraid, also are afraid of being found out by others, (and) why? It’s just (because) first of all, (the patient) is afraid of being discriminated. (And) second, (the patient is) just afraid that it will affect some of his life, work, and studies. Hmm! If (they) are only co-workers, (the patient) doesn’t really want to tell others (the patient’s) own matters, because (he) is also afraid that it’ll be spread to the entire factory! (D05)

A climate of poor public-opinion and trust in the medical profession

因为现在国内医患关系比较紧张，社会舆论让大家都觉得看病贵，钱都被医生赚了，其实钱都没有到医生这里来。还是要从国家的制度体系进行改善，还有患者的自己心理体验。

> Currently, the relationship between doctors and patients is somewhat tense. The public opinion makes people think it requires a high cost for getting medical treatment, and all

the money is earned by doctors. Actually, it’s not the truth. It should be improved from the national system. (D07)

但是他有时候觉得医生不靠谱，情况没有改善。我有遇到一个病人在其他社康看过治疗失眠，但后面他家属补充有在内地的医院看过精神科。当时他就要求说吃中药，我没有给他吃，我说那个意义不大，让他去精神科医院看一下，后来我追踪发现最终的他不愿意去，即使他家属很想带他去，后来他跑了。

Sometimes patients think physicians are unreliable because their situation has no improvement. I saw a patient who went to other community health service center for the treatment of insomnia before, and the patient’s family said they also went to psychiatry department of one hospital in mainland China. At that time, the patient required to take traditional Chinese medicine. I didn’t prescribe it to him/her and told him/her it made little sense. I recommend the patient to go to psychiatric hospital, but I found that he/she was unwilling to go after follow-up. His/her family wanted to take him/her to the hospital, but he/she ran away. (D02)

Patients uncompliant with doctor recommendations for referral or treatment

检查的困难来自病人的不配合。因为有焦虑、抑郁的病人，他的情绪会波动很大。你给他提一些建议，让他去做一些检查，他会拒绝或者是干脆不做。有些检查不可能在社康就做让他去打医院，有些病人会觉得麻烦或者是不方便，就不想做让我们开点药就算了。

Difficulty of examinations is noncompliance of patients. Emotional fluctuation of patients with depression or anxiety is usually great. They will refuse or not do it when you advise them to receive some examinations. We may recommend them to go to hospitals for receiving some examinations because they can't be conducted in community health service centers. Some patients think it’s troublesome or inconvenient, and just want us to prescribe some drugs for them. (D15)

有的人觉得抑郁症不是什么问题，他也认可，但就是不愿意吃药。这一片区的人文化程度比别的社区高一些，所以对于抑郁症的了解还算比较充足。有时候病人说他吃这个药不大舒服的样子让换一个药。还会说可能有一个别的什么药，他自己会查这一方面的信息。

Some patients recognize that they have depression, but they don’t consider it a problem and are unwilling to take medicine. People live in this community have received higher education, so they know better about depression. Sometimes, people feel uncomfortable after taking a medicine, so they search for relevant information to switch to another. (D10)

Trust in the doctor is a key component for treatment success

如果他很积极的、依从性很高的、他很信任我的，是及其帮助的作用，病相对来说好的快一些。他也可以不信任、他说我没有精神病，或者他对这个病的理解程度不够，或者是他潜意识的排斥，我就没有办法治疗下去，这就是阻碍的地方。

If the patient is positive, his/her compliance is high and he/she trusts me, it will be quite helpful, and the disease can be cured quicker relatively. If the patient doesn't trust me or he/she doesn't understand the disease well, or he/she rejects it subconsciously, I will have no way to treat it. This is the obstacle. (D08)

TDF Domain 11: Behavioural Regulation

Require depression-specific polices with drug reimbursement

可能会要很久很久以后了。因为现在能够引起大众重视的就是重型精神障碍，所以在这一块会有更多的政策支持。像精神障碍的药物是免费，经过申请会给你的，但是抑郁症没有。

A long time might be in need. Now we pay much attention to severe mental disorder, so there is corresponding policy support, and drugs for mental disorder are free after patients apply. However, there is no such policy for depression. (D10M)

医保没有意识到这一个问题，我们国家的医保主要停留在身体上的疾病。虽然精神的疾病有政策的，但对于抑郁是没有，除非是重度精神病的是有免费服药、体检的政策。

Medical insurance hasn't realized this problem yet, and the medical insurance of our government mainly cover physical diseases. Although there are relevant policies for psychiatric diseases, no such policy available for depression. However, patients with severe psychiatric diseases can get free medicine and physical check. (D09)

Require Dr-incentivisation (like psychosis policies)

啊，因为我们GP是没有任何药物的。但是我能够诊断出他肯定是有幻想、幻听，这些是属于精神分裂症的。已经严重了。我们只能够转诊，转到那个专科医院。就是建议他去深圳的精神病科医院去看。但是我们要上报。就是说要登记，这个小伙子身份证、电话号码、原籍在哪里。因为深圳是移民城市，很多人不是在深圳土生土长的。要是把这个报告写上去，并且政府还鼓励的。如果GP发现一例，还有一定的 鼓励你发现、上报。

Ah, since we GP don't have any medicines {specialized in treating mental illness}. But I can diagnose that he surely has illusions and auditory hallucinations which belong to schizophrenia. (He was) already seriously (ill). We could only refer (him) to that specialized hospital. It's like suggest that he go to the psychiatric hospital in Shenzhen. But we need to report (this case). It's like to register, this young man's identification card, telephone number, (and) where is originally from. Since Shenzhen is a city of immigrants, (and) many people are not native in Shenzhen. If (we) report this (case), the government will also encourage (us GPs). If the GP discovers one case (of mental illness), there will be certain money (reward) to encourage you to discover and report (more cases). (D04)

"One Psychiatric Doctor per Community Health Centre" facilitates passing down & cross-referral

一个精神、我们每一个社康的话必须要有一个精神科医生。然后现在也、我们的卫生、卫计委也开设了一个精神科转岗医生的培训。要培训全科医生去拿证上岗。然后呢……，已经意识到了。

There must be a psychiatric doctor at every of our community-based clinics. Then now also, our Health and Family Planning Commission has also set up a training

> program for (GPs) to transfer into psychiatrist, to train the GP to achieve the certificate and work on (the psychiatrist) post. Then…, (the government) has already had the awareness (that mental health requires more attention). (D01)

都可以谈。现在有的就是……如果实现确诊了的病人，比如说现在有的、像康宁医院，它确诊了的病人它会弄到那个系统里面去，然后……我们这里公卫医、那个管精防的医生也能看得到这个病人。它会、叫那个那个，我们叫下转！对，叫下转！我们叫下转给我们的精神卫、卫、管精神卫生的医生。

> Both can be talked about. What's existing now is…if the patient has already been realized the confirmed diagnosis, for example, what's existing now (is), like Kangning Hospital, the diagnosed patients will be recorded to that system, then…our public health doc-, that doctor in charge of psychiatric prevention can also see this patient. It (the system) will, it's called that that, we call pass down! Yes, it's called pass down! We call that passing down to our mental heal-, health, the doctor who is in charge of the mental health (area). (D06)

对，也有过医生专门做过心理咨询这一块的训练，比如说我并不擅长处理这个、这个又不是我的老病人，可是我知道你可能遇到这方面的问题，刚好那个有精神资格的全科医生也不在，或者说又不至于需要专科医生看的时候的我就会让我同事来看。

> Yes. Some doctors in our centre received training about psychological counselling before. For instance, if the patient is not familiar to me, and I'm not good at the treatment of the disease, I may ask my colleague for consultation when the general practitioner with qualification is not there or when it's unnecessary to invite the specialist for treatment. (D02)

Establishment of dedicated mental health department at local hospital

啊，对！现在要成立一个精神科，就是为了、第一个，它有、它有几个作用，就是说因为他们也知道我们基层的社康的医生可能对这一方面的知识比较薄弱，那专门设立了这一个科，我们还建了一些微信交流群。比如说我们碰到一些什么患者，自己不知道怎么处理或是怎么，我们可以在那个群上问那个主任，问那个主任。然后他可能会给我们一些建议。如果我们自己解决不了，我们可能会转给他。因为怎么说呢？因为、因为你，因为我们是属它医院集团管的嘛，然后如果我们转上去的话，我们还可以跟他那个接诊的医生还有一定的交流。你转到康宁医院的话，你很难追踪到那个患者的信息。啊！

> Ah, yes! Now (the Group) will establish a mental health department, just for, firstly, it has, it has a few functions, (and the first one is) just because they {the Group management} knows that the doctors from community-based clinics who are at the basic level of healthcare might not have solid knowledge in this {mental health} area, so (the Group) specially established such a department (as well as) some Wechat communication groups. For example when we {the GPs} come across some patients, (and) do not know how to deal with it by ourselves, we can consult that Director in that chat group, ask that director. Then he might give us some advice. If we can't solve it ourselves, we might refer (the patient) to him. Because, how to put it? Since, since you, since we are under the management of the Hospital Group, plus if we make a referral up (to the Group), we can still have certain communication with the doctor who accept the referral. (If) you {the GP} make a referral to Kangning Hospital, you will have a hard time track that patient's information {progress}. Ah! (D05)

Review of “Five in One Policy”

非常困难，而且很不配合。为什么要现在五位一体，第一我们医生自己不安全、第二患者自己很排斥，他有保护自己的隐私，他也不希望别人知道他有这个病。第三我们有时候会通过一些奖励性的措施，比方说给他们去送一些礼品，例如米、油之类，让他们配合一下，过来免费全身体检。他们还是不愿意配合。他们觉得是自己的隐私，但他们的态度也是可以理解的，他不想让那么多的人知道。管理的话还是很难做的，因为这不是一份好差事。我们精神科的医生都不想做了，老是跟我说要换人。

> It’s extremely difficult and patients don’t cooperate with us so well. Why the policy of “five-in-one” is necessary? Firstly, physicians feel unsafe. Secondly, patients reject it themselves. They want to protect their privacy and don’t want others know that they have the disease. Thirdly, sometimes we take some incentive measures such as giving patients some gifts of rice and oil to make them cooperate with the general physical examinations. Their attitude is still understandable if they are still unwilling to cooperate and think it’s their privacy. Therefore, the management for patients like this is difficult. Many physicians in our psychiatry department want to resign. (D12)

Stronger health promotion on world mental health day

深圳在全国走在前面的，因为政策比较多，包括对于小孩儿的孤独症的筛查、对于产妇的筛查、对于老年人的抑郁量表已经意识到了，但是他这个是一个项目形式来做，作为一个前期的工作，并没有真正放在临床上实施过。

> Shenzhen has already been a leader in this field, as this city has established many policies, including screening children autism, maternal depression and elderly depression. However, it’s the preparatory work of a program, and it hasn’t been conducted in clinical practice. (D09)

Internet counseling a potential opportunity

我有个朋友，有个护士，她也考了心理咨询证，她就是在网上做心理咨询师。就是按那种网上给钱，就是给那种虚拟的币还是什么币，我也不知道。因为什么呢？因为她是护士，她的职业生涯即将要到头，她不想老来了以后就老无保障。像我们医生，我们老了以后还可以返聘，还可以继续行医。但是她护士老了，她就没那个了。所以她就想等她快退休的时候就改行做这个心理咨询师。所以她就在网上做。.

> I have a friend, a nurse, she also holds the psychological counselling certificate, (and) she is an online psychological counsellor. (She is) paid online by those virtual money or something, I don’t really know….. Why is that? Because she’s a nurse, (and) her career is reaching its end (and) she doesn't want to end up with no (economic) back up. As doctors, we could still be reemployed and continue to see patients when we are old {retired}. But she doesn’t have that when she’s old as a nurse. So she’s thinking when she’s about to retire she could change career to be a psychological counsellor. So she’s doing that online. (D03)

TDF Domain 12: Reinforcement (needs)

Improved doctor training with special instruction in mental health

首先第一个在这个专业上提供一些培训，除了理论培训，还要有一些类似于角色扮演之类让我们能更深入的掌握，而不是表面性的东西。其实我们也有学这一门课程，但是都是特别表面的东西。最好到专科里进行实践培训。一个理论、一个实践相结合的培训。

> Firstly, some relevant training needs to be provided. Besides theoretical training, we also need role play for better understanding about it. Actually, we learned relevant knowledge before, but it was extremely shallow. It will be the best for us if there is practical training. The integration of theory with practice is important. (D07)

Access to Western medicine and an improved consulting environment

就是有想要用的药我们也能开，要有足够的时间，诊室的环境要安静一些。如果一大堆的人来看病根本就没有办法开展。

> It is the best if we can prescribe corresponding drugs, have enough time and a quiet consulting room. It's impossible to work well with a lot of patients in the consulting room. (D17)

第一需要一个有效的治疗。一个非常有效、副作用又小，相对来说适合大部分情况的药物对病人的痛苦会少一些.

> Firstly, we need effective treatment. If there is effective treatment with mild side effects, most patients can suffer less pain. (D14)

一个是培训和药品要够。社康没有药品，我们怎么能治疗呢？

> Firstly, sufficient training and drugs. It's impossible for us to treat depression without drugs. (D08)

Improved health literacy

然后呢，再就是我个人觉得可能是，政府要在这个、这个公共宣传方面、教育方面可能要多一点资源。比如说我看澳大利亚这边的非常多的这个网站，都是跟、各个方面的疾病，都是非常好的一些公共传播的一些知识。但是国内这方面就非常少。病人就算知道了，他也只能去百度上去看一看。但百度上面的很多咨询都是没有用处的。垃圾信息。对、对，没有、没有特别专业的信息给病人一些……或者让大家认识到这、这其实是很普、很普遍的一个问题，这不是一个很难开口的一个问题。就大家都知道了这样一个事情，可能他就寻求帮助的机会……或者他就能说出来，就、就、就这个。这样的一个知识系统比较少。对，你这、因为你不可能一直关着这个盒子在里面发酵，发酵到一定的程度肯定就会要爆发。所以我就觉得是一个公众对这种疾病的认知的问题。最关键是这个认知的问题。那么大家都认为它像感冒发烧一样的，这个就是一个人呢他除了身体器官的问题，他还有其他的这个、这个问题的话，大家都可以很正常的，你也有，我也有，就不会有歧视。嗯，不会有歧视，那、那就可以很open地讲，这样的一个事情。反而我觉得不太容易有这样的问题了。

> Then, the next I personally think maybe, the government needs to allocate a bit more resources in the area of this, this publicity, (and) education..For example, I have observed in Australia that there are very many this (type of) websites (which) are (about) all kinds of diseases containing very good public promotion of knowledge. But it is very little in the country{China}.Even if they patient is aware of (his illness), he can only have a search in Baidu. But many information in Baidu is useless. Trash information. Yes, yes, there is no, there is no very professional information that could provide (useful advices) to the patient…or could help

people realize this, this is in fact a very com-, very common issue, (but) not a very embarrassing problem. If everyone is aware of such a thing, then maybe he (will have bigger) chance to seek for help… or he will be able to speak it out, and it's like this. Such a knowledge system is rather limited. Then I…I think maybe, the most critical thing is still like…what they said, that you need to open this box. Yes, you this, because you can't be locked inside the box and ferment, (and) ferment to certain degree and (you) will surely explode.So I think this is an issue about public awareness of this disease. The key is the awareness. Then (if) everyone thinks it's the same as (having) a cold or fever, (and) this is just for a person, in addition to having problems with his physical organs, he will have other this {mental illness}, this problem, (then) everyone will be able to (treat it) normally, you have this, (and) I also have this, (and) there won't be any discrimination. Hmm, there won't be any discrimination, then, then (people) can be open to speak about such a thing. Instead I don't think it easily causes such problems. (D01)

TDF Domains 13 & 14: Intentions and Goals

The care of depression patients to be more strongly prioritized

我觉得应该。这一部分的病人等待看诊的时间久了或者本来对医生久抱着不好的态度，如果照顾不好的话会容易烦躁，就会引起不和谐的事情造成投诉或者对我们社康的满意度造成影响。另外在社康就诊的绝大部分的病人病情相对比较轻的，所以其他人可以等待，而这种的病人因为情绪有障碍，可能会由于等得不耐烦、等久了走了，就会造成病情的延误。

I think there should be a health priority. Patients like this get easily annoyed if they have to wait for the diagnosis for a long time or their attitudes towards physicians are not good or they are not well cared for. It's easy to cause disharmony and complaints of dissatisfaction with our community health service center. Besides, most patients who come to community health service centers for treatment only have mild disease. Perhaps it's acceptable for general patients to wait for a long time, but patients with emotional disorder can't wait for such a long time and then they may go away, which will cause the delay of disease. (D13)

Good psychological health is an important component for quality of life

这样说吧，我觉得在看病的时候，不管他是焦虑的、抑郁的还是正常的，都要关注他心理健康方面的问题。如果是有发现就及时进行干预。我看病的时候就会更注重心理健康方面的问题，因为这样于一个人的生活质量、心理健康会更好、更有帮助。

In my perspective, we need to pay attention to patients' psychological health, no matter whether they suffer anxiety or depression or not. We need to intervene when discovering such cases. I pay more attention to patients' psychological health, as good psychological health is good for their quality of life. (D15)

Timely management of depression prevents suicide

是。抑郁症是隐藏得比较深的问题，如果没有及早的解决会造成很不好的东西，甚至是会产生悲剧。而且现在很多人觉得这不是问题，所以比较危险。

Yes. Depression is a hidden disease and it will cause bad consequences if it's not solved timely, and even cause tragedy. Besides, many people now think it's not a problem. Therefore, it's somewhat dangerous. (D11)
